# Retrieval-Based Diagnostic Decision Support: Mixed Methods Study

**DOI:** 10.2196/50209

**Published:** 2024-06-19

**Authors:** Tassallah Abdullahi, Laura Mercurio, Ritambhara Singh, Carsten Eickhoff

**Affiliations:** 1 Department of Computer Science Brown University Providence, RI United States; 2 Departments of Pediatrics & Emergency Medicine, Alpert Medical School Brown University Providence, RI United States; 3 Center for Computational Molecular Biology Brown University Providence, RI United States; 4 School of Medicine University of Tübingen Tübingen Germany

**Keywords:** clinical decision support, rare diseases, ensemble learning, retrieval-augmented learning, machine learning, electronic health records, natural language processing, retrieval augmented generation, RAG, electronic health record, EHR, data sparsity, information retrieval

## Abstract

**Background:**

Diagnostic errors pose significant health risks and contribute to patient mortality. With the growing accessibility of electronic health records, machine learning models offer a promising avenue for enhancing diagnosis quality. Current research has primarily focused on a limited set of diseases with ample training data, neglecting diagnostic scenarios with limited data availability.

**Objective:**

This study aims to develop an information retrieval (IR)–based framework that accommodates data sparsity to facilitate broader diagnostic decision support.

**Methods:**

We introduced an IR-based diagnostic decision support framework called CliniqIR. It uses clinical text records, the Unified Medical Language System Metathesaurus, and 33 million PubMed abstracts to classify a broad spectrum of diagnoses independent of training data availability. CliniqIR is designed to be compatible with any IR framework. Therefore, we implemented it using both dense and sparse retrieval approaches. We compared CliniqIR’s performance to that of pretrained clinical transformer models such as Clinical Bidirectional Encoder Representations from Transformers (ClinicalBERT) in supervised and zero-shot settings. Subsequently, we combined the strength of supervised fine-tuned ClinicalBERT and CliniqIR to build an ensemble framework that delivers state-of-the-art diagnostic predictions.

**Results:**

On a complex diagnosis data set (DC3) without any training data, CliniqIR models returned the correct diagnosis within their top 3 predictions. On the Medical Information Mart for Intensive Care III data set, CliniqIR models surpassed ClinicalBERT in predicting diagnoses with <5 training samples by an average difference in mean reciprocal rank of 0.10. In a zero-shot setting where models received no disease-specific training, CliniqIR still outperformed the pretrained transformer models with a greater mean reciprocal rank of at least 0.10. Furthermore, in most conditions, our ensemble framework surpassed the performance of its individual components, demonstrating its enhanced ability to make precise diagnostic predictions.

**Conclusions:**

Our experiments highlight the importance of IR in leveraging unstructured knowledge resources to identify infrequently encountered diagnoses. In addition, our ensemble framework benefits from combining the complementary strengths of the supervised and retrieval-based models to diagnose a broad spectrum of diseases.

## Introduction

### Background

Identifying an accurate and timely cause for a patient’s health problem represents a challenging and complex cognitive task. A clinician must consider a complex range of composite information sources, including the patient’s medical history, current state, imaging, laboratory test results, and other clinical observations, to formulate an accurate diagnosis. Diagnostic errors are a leading cause of delayed treatment, potentially affecting millions of patients each year. Research suggests that these errors contribute to 6% to 17% of adverse events [1].

Studies [2,3] have shown that, rather than relying on a single physician for a final diagnosis, obtaining recommendations from multiple physicians increases diagnostic accuracy. To improve the diagnostic process while maintaining economic feasibility, different variants of automated assistants, also known as diagnostic decision support systems (DDSSs) and symptom checkers, have been introduced [4]. Early DDSSs [1,5] were driven by structured databases that maintain information about diseases and other medical information in a structured form. Although promising, these systems have yet to be highly successful for several reasons, including limited accessibility, poor flexibility, and scalability issues [6,7]. Hence, the traditional DDSS is gradually being replaced by machine learning and deep learning models.

Recent studies [8-13] highlight the importance of electronic health records for supervised machine learning algorithms in health care. These algorithms use the electronic health record of a patient as input to predict their diagnosis. However, supervised model development has been limited to a select number of diseases with higher prevalence and extensive documentation due to the availability of large amounts of labeled data. As a result, infrequently occurring diagnoses remain poorly studied. In real-world diagnostic scenarios, physicians are faced with the challenge of identifying the correct diagnosis from a plethora of possibilities. Therefore, a system that considers a broad range of diagnoses, including rare conditions, is desirable for improved diagnostic accuracy. However, recent studies [14,15] demonstrate that traditional supervised learning models are challenging to use in such scenarios due to their reliance on large, labeled data sets with many examples per diagnosis. However, most clinical cohorts exhibit imbalanced class distributions, characterized by a long-tailed pattern [15,16] in which certain diagnostic classes represent most training samples whereas others exhibit few or even 0 data points. In such scenarios, most traditional supervised models overfit the majority class, resulting in poor performance for the minority classes. As such, large labeled data sets may not be a straightforward solution for achieving an efficient supervised classifier that supports diverse diagnoses.

In response, researchers have leveraged a technique called transfer learning, which is a widely used method for building classifiers that enables generalization to classes with limited labeled data. A common transfer learning technique involves fine-tuning pretrained models—models trained on large and diverse data sets—on a smaller, domain-specific corpus to enhance model performance. However, the effectiveness of this approach still relies on the size of the data set available for fine-tuning. Zero-shot learning and few-shot learning [17,18] represent promising alternatives for fine-tuning large models with limited labeled data. In zero-shot learning, the model can classify samples from classes without labeled training data. Few-shot learning requires at least one labeled example per class to enable the model to make accurate predictions. Although some studies [19,20] have shown that pretrained language models have zero-shot and few-shot learning capabilities, their performance remains inferior to that of models trained on extensive labeled data. While zero-shot and few-shot approaches have demonstrated success in the vision domain [21,22], their application to language models remains an ongoing area of research.

Leveraging external knowledge resources can improve predictive performance, especially with a limited training sample size, as shown in previous work by Prakash et al [7] and Müller et al [6]. Classical information retrieval (IR) systems can use a vast collection of resources for various applications with low computational complexity and no need for labeled data. In the medical setting, studies [23-25] and competitions such as the text retrieval conference (TREC) clinical decision support track [26] have focused on developing and evaluating IR systems to support clinician decision-making. Typically, these IR systems have been applied to biomedical literature retrieval to aid in clinical decision support. However, these systems can also be adapted for other downstream clinical tasks. For example, Naik et al [27] trained a model to predict patient admission outcomes (ventilation need, mortality, and length of stay) by integrating relevant medical literature with patient notes, leaving an open question of how IR systems would fare in directly predicting the underlying diagnosis. Therefore, our study applied IR techniques to perform literature-guided diagnostic prediction.

### Objectives

We introduce “CliniqIR,” a novel clinical decision support algorithm that uses an IR system to match a patient’s medical record to a specific diagnosis from a large pool of possible diagnoses. Our study aimed to improve the current state of predictive modeling and diagnostic decision support for a broad range of diagnoses regardless of their training data availability. By using clinical text records and external knowledge sources, including the Unified Medical Language System (UMLS) Metathesaurus [28] and PubMed abstracts [29], we demonstrated that “CliniqIR” successfully generalizes to less common diagnostic categories with heavily skewed data distributions. Our work also shows CliniqIR to be highly adaptable, allowing for easy integration with any IR system. This flexibility ensures the model’s ability to adapt to available resources and work across various retrieval methods.

**Figure 1:**
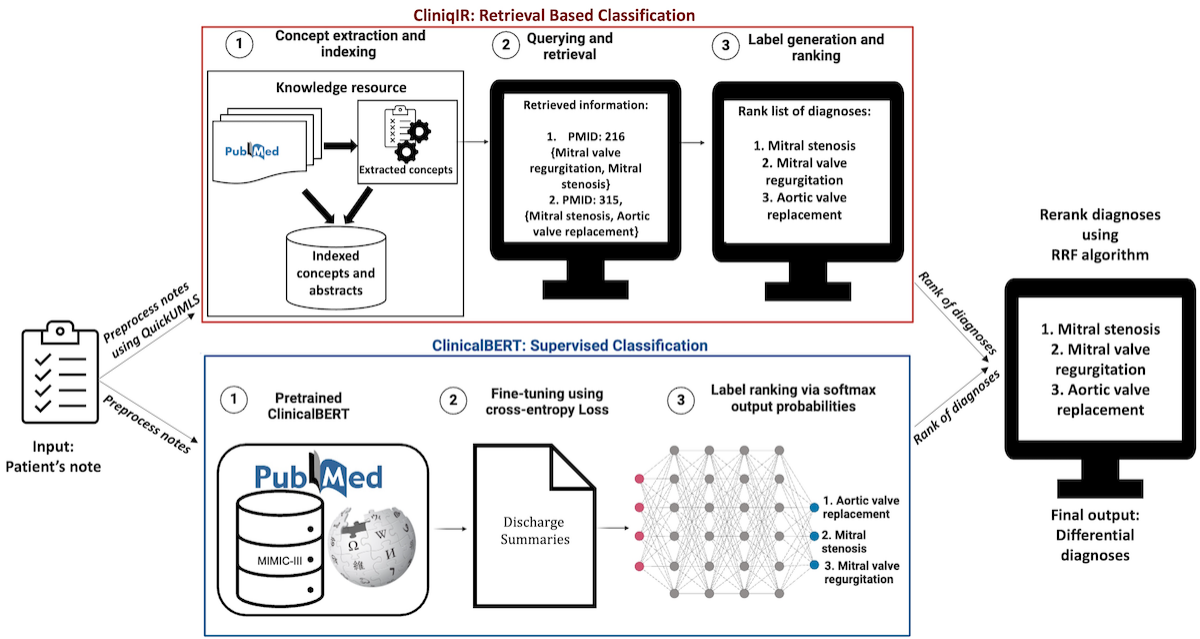
CliniqIR and Clinical Bidirectional Encoder Representations from Transformers (ClinicalBERT), classify patient notes and generate ranked lists of potential diagnoses. The reciprocal rank fusion (RRF) ensemble reranks the lists from both models to provide clinicians with a more accurate final ranking of differential diagnoses to aid the diagnostic process. MIMIC-III: Medical Information Mart for Intensive Care III; PMID: PubMed ID.

To assess CliniqIR’s ability to predict diagnoses with no available training samples, we evaluated its performance on the DC3 data set [30]. We compared its performance to that of pretrained clinical models in a zero-shot setting, and our results showed that CliniqIR has the capability to recognize a broad spectrum of rare and complex diseases without relying on labeled training data. We also compared the performance of CliniqIR with that of supervised fine-tuned pretrained biomedical large language models and found that supervised models have limitations when used on highly imbalanced data, especially for diagnoses with limited training samples. Then, we leveraged an ensemble strategy combining CliniqIR and a fine-tuned Clinical Bidirectional Encoder Representations from Transformers (ClinicalBERT) to make predictions for a wide range of diagnoses that include frequent and infrequent conditions, summarized in Figure 1.

Our study highlights the valuable synergy between retrieval-based systems and supervised learning models, showcasing how their combination can achieve state-of-the-art performance, particularly in data sets characterized by a long-tailed distribution. This finding holds significant promise and offers new avenues to address the challenges of imbalanced data in various domains.

## Methods

### CliniqIR: The Retrieval-Based Model

#### Overview

We present CliniqIR, a novel literature-guided system that maps a patient’s note to a specific diagnosis. By leveraging unlabeled external knowledge sources, CliniqIR uses an IR system to classify a wide range of diagnoses without relying on the availability of notes for each individual diagnosis (labeled training data). As a result, CliniqIR represents a valuable disease classification tool when labeled training data are limited or unavailable.

**Figure 2:**
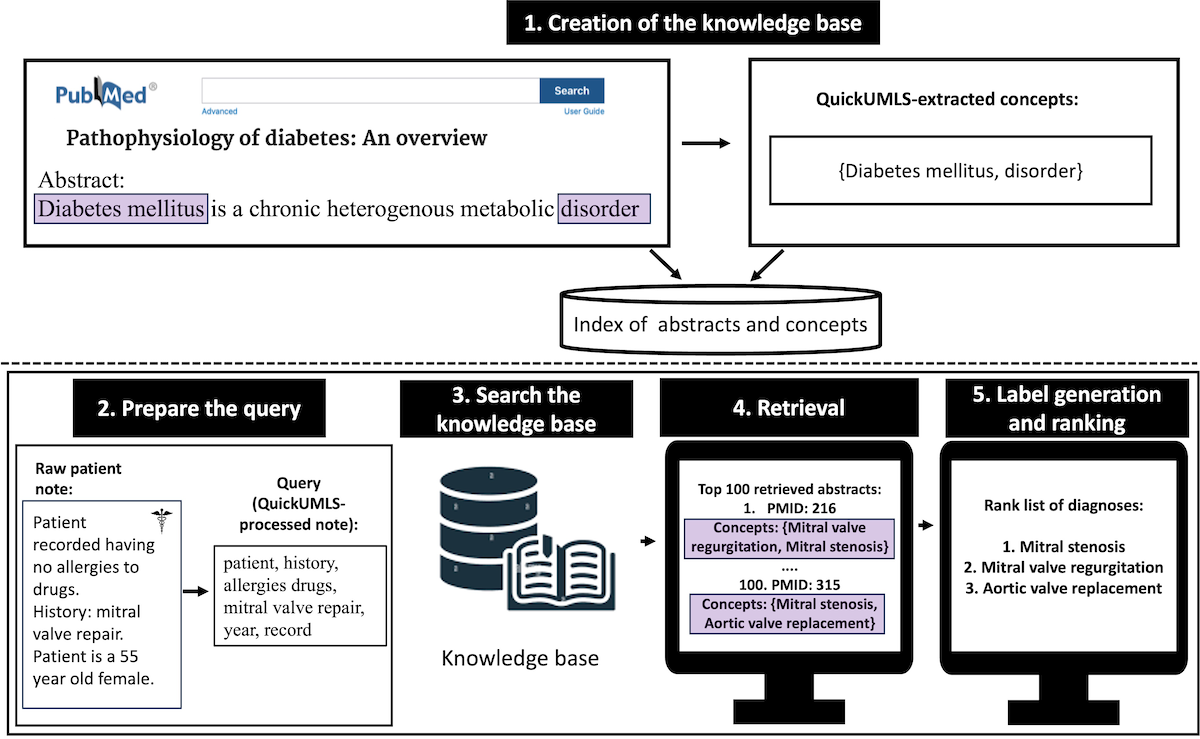
Overview of CliniqIR, the retrieval-based clinical decision support system. PMID: PubMed ID.

An overview of our method is shown in Figure 2. The backbone of CliniqIR is its knowledge base. Once the knowledge base is built, we can query the system to provide a list of probable diagnoses. In this study, a clinical narrative with a patient’s medical history or summary was preprocessed and treated as a query. To make inferences given a patient’s clinical note, as a preprocessing step, we first used QuickUMLS (Soldani and Goharian [31]) explained in the *Knowledge Extraction Using QuickUMLS* section, to extract medical keywords from the note to obtain a query. Next, we fed the query (preprocessed note) to the retrieval system, which returned a list of matching relevant PubMed abstracts alongside the medical conditions mentioned in each abstract. Afterward, we selected the top 100 items from the list and then computed the frequency of each concept across the list of abstracts. Finally, the model returned a list of concepts ranked according to their term frequency–inverse document frequency (TF-IDF) defined in equation 1. The list returned was similar to a medical differential diagnosis (a ranked list of possible diagnoses that could cause a patient’s illness). The medical condition with the highest TF-IDF score was predicted as the most likely diagnosis. We provide a detailed description of the individual processing steps in the following sections.

#### Concept Extraction and Indexing

We extracted unique medical concepts (conditions) from each PubMed abstract using QuickUMLS, described in the *Knowledge Extraction Using QuickUMLS* section. Medical concepts included diseases, symptoms, or any information about a medical procedure. Subsequently, we built the knowledge base of the retrieval system by indexing each abstract and its corresponding article title, article ID, and a concept dictionary that contained all the unique concepts mentioned in that abstract. Indexing involves storing and organizing data to enable efficient IR at search time. Using the index of PubMed abstracts, the model inputs a patient’s notes as a query and returns relevant information from the indexed abstracts as an output. Figure 2 provides visual details.

#### Querying and Retrieval

Once the index was built, we submitted queries to the retrieval system. The *Retrieval System Implementation* section provides more details. After we submitted a query, the system returned a list of abstracts and their corresponding attributes (dictionary of concepts, article title, and article ID number) ranked according to query relevance. For each query, we selected the top 100 abstracts because the top few documents are most likely to contain relevant query information.

#### Label Generation

After the querying operation, we focused on the extracted concepts of the top 100 abstracts. The previous retrieval phase can potentially return multiple abstracts that contain similar information in response to a given query, resulting in concept dictionaries of ≥2 abstracts containing similar concepts. Multiple occurrences could indicate the relevance of a concept across abstracts. To account for such duplication, we calculated each unique concept’s recurrence, or term frequency (TF), across the list. The TF of a concept across a list of abstracts would be 1 if it appeared in only 1 abstract. If it appeared in 2 abstracts, its TF would become 2, and so on. Calculating the recurrence of concepts across the top-100 list resulted in a new list that contained medical concepts and their TFs. These medical concepts were regarded as labels and used for classification purposes. Thus, each unique concept became a potential diagnosis, and the TF of each concept is subsequently used for ranking purposes in equation 1. Textbox 1 describes the concepts the model returned (in no order of importance) after the retrieval stage given a set of queries processed using QuickUMLS. The list was filtered for a simple illustration. As mentioned previously, concepts are biomedical terms that include symptoms, signs, and diseases, among other things. On the other hand, a diagnosis could represent a disease, an injury, a neoplastic process, or a medical term describing a condition a patient is experiencing. Therefore, to account for a wide range of possible diagnoses, we kept all concepts in the label generation phase, and we considered a concept as a diagnosis when it matched the ground truth. Therefore, in this paper, we use *concepts* and *diagnoses* interchangeably.

**The output returned by the retrieval-based model (CliniqIR) given a query.**

**Query and concepts retrieved (labels)**

- Abdominal pain, bloating, rectal bleeding, weight loss, anxiety, disruptive thoughts, and suicidality: “generalized anxiety disorder,” “panniculitis,” “chronic abdominal pain,” “Burkitt’s lymphoma,” and “Whipple’s disease”
- Chest pain, radiation to neck, dyslipidemia, lung crackles, bradycardia, and ST elevation: “acute myocardial infarction,” “acute coronary syndrome,” “coronary artery disease,” “myocardial ischemia,” “myopericarditis,” and “myocardial infarctions”
- Night sweats, abdominal pain (pleuritic), nausea, loose stools, lymphadenopathy (inguinal), plaques, leucopenia, neutrophilia, and elevated (Angiotensin converting enzyme) ACE: “sarcoidosis,” “lymphomas,” “lymph node,” “tuberculosis,” “lupus erythematosus,” “Rosai-Dorfman disease,” and “Kikuchi-Fujimoto disease”

#### Ranking and Predictions

It is important to note that our model differs from traditional classification schemes. In our case, the observed mappings between patients’ notes and ground-truth diagnoses are not provided for learning purposes. Therefore, a list of relevant diagnoses (a subset of the retrieved concepts) must be generated independently for each query. However, as the diagnosis list is not generated based on ground truth, it may contain information that is not relevant to the data set to be evaluated. For example, given a data set with 3 possible ground-truth diagnoses—*lymphoma*, *coronary artery disease*, and *gastroenteritis*—the model might return concepts such as *coronary artery disease*, *myocardial infarction*, and *chest pain* in the label retrieval phase for a query whose ground truth is *coronary artery disease*. To address this and ensure a fair comparison with other classification models, we filtered the retrieved concepts during the evaluation and only kept diagnoses that were part of the ground truth. Therefore, in the aforementioned example, we filtered out *myocardial infarction* and *chest pain*. Then, we assigned ranks to the remainder of the diagnoses in the list using the TF-IDF function shown in equation 1:


TFIDF(c,a,d) = TF(c,a).IDF(c,d) **(1)**

#### Knowledge Resources: PubMed Abstracts

Over the years, research in predictive modeling for diagnostic decision support has witnessed enormous success in transfer learning, particularly where a model leverages an auxiliary data source (often a knowledge base) to perform several predictive tasks. Some studies [7,22,32] have used resources such as Wikipedia and PubMed [29] to create systems that perform classification tasks or retrieve useful articles with specific information. In contrast, most early DDSSs [1,33] were built on structured knowledge bases; however, most computable knowledge bases are not freely accessible.

Inspired by previous research, we used abstracts from PubMed articles as an unstructured collection of knowledge resources to guide the prediction of diagnoses for all our experiments. An abstract may contain information about a specific condition, its signs, or its symptoms. Some abstracts include medical case reports, whereas others may contain information about a medical device. To build a retrieval system grounded in reliable information, we leveraged the vast collection of abstracts in the PubMed database. PubMed, maintained by the National Library of Medicine, houses >33 million citations for biomedical literature, encompassing life science journals and books dating back to 1946. However, the number of abstracts available per condition varies considerably. Therefore, for our core experiments, we implemented a 100-abstract inclusion threshold for diagnoses (Multimedia Appendix 1).

#### Knowledge Extraction Using QuickUMLS

##### Overview

QuickUMLS [31] is an unsupervised medical concept extraction tool that detects mentions of medical entities such as diseases, symptoms, and other medical concepts from unstructured text. Given a document, QuickUMLS matches each possible token in the document against concepts in the UMLS [28]. In this study, we used QuickUMLS for 2 different purposes.

##### Extraction

**Figure 3:**
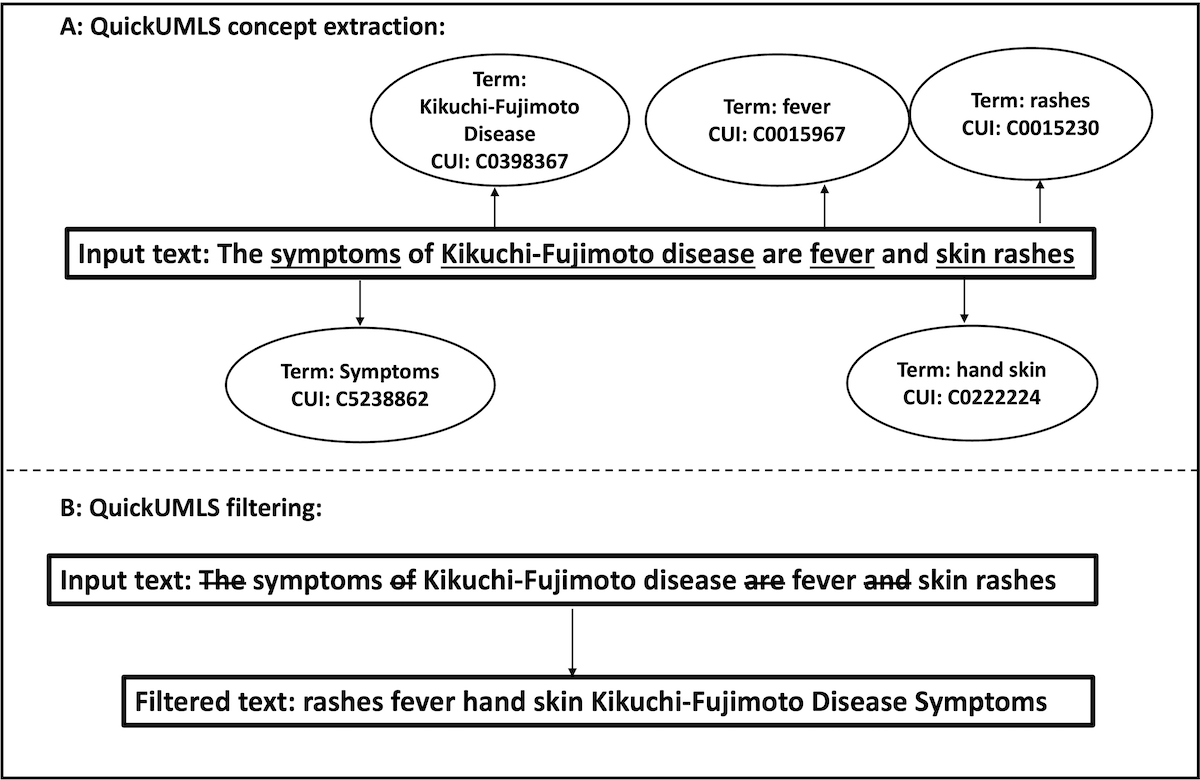
Outputs from the QuickUMLS tool developed by Soldani and Goharian [33] showing: (A) a graph of extracted concepts and their concept unique identifier (CUI) for a specific input text; the underlined texts are considered important words, and their corresponding Unified Medical Language System terms and CUIs are returned, (B) a query processing pipeline. Each text marked with a strike-through is filtered out to obtain a query.

We used QuickUMLS to extract unique biomedical concepts from each PubMed abstract. A concept can be any medical term, including a diagnosis or symptom. As shown in Figure 3, each biomedical term in a text is a concept with a corresponding unique alphanumeric identifier (concept ID) in the UMLS vocabulary. We kept all the concepts associated with each abstract in a dictionary.

##### Filtering

We also used QuickUMLS as a data preprocessor to filter out noisy, uninformative, and nonclinical terms, such as stop words, from a patient’s clinical note, resulting in a query that contained only medical terms. Citing the input text example in Figure 3, the outcome of the filtering operation was “rashes fever hand skin Kikuchi-Fujimoto disease symptoms.” This filtering step is equivalent to keeping only the QuickUMLS-recognized medical terms and concepts.

### Retrieval System Implementation

#### Overview

CliniqIR is designed to be highly adaptable to arbitrary IR systems. This flexibility ensures the model’s ability to work across various retrieval methods, adapting to the resources available. In this study, we performed experiments on a sparse and a dense retriever.

#### Sparse Retriever

We built our knowledge base by indexing PubMed abstracts and their concepts using Apache Lucene (Apache Software Foundation [34]), which enables users to search this index with queries ranging from single words to sentences. The relevance of an abstract to a query is determined by a similarity score, with Lucene’s default “BM25” [35] function estimating the best-matching abstract.

#### Dense Retriever

Unlike sparse retrievers, which represent queries as word frequencies, dense retrievers capture the semantic meaning and relationships within the text using dense embedding vectors. This allows for retrieval based on similarity, usually calculated through maximum inner-product search. To implement this approach, we leveraged the Medical Contrastive Pre-trained Transformers (MedCPT) [36], a state-of-the-art biomedical retrieval system in a zero-shot setting using its default parameters. Section S1 and Figure S1 in the Multimedia Appendix 1 provides details on the parameter settings for both retrieval systems.

### Pretrained Transformer Models

In this study, we used 2 well-known methods, namely, supervised fine-tuning and zero-shot learning, to harness the benefits of transfer learning from 6 pretrained clinical and biomedical language models. The models we used are ClinicalBERT, PubMed Bidirectional Encoder Representations from Transformers (PubMedBERT), Scientific Bidirectional Encoder Representations from Transformers (SciBERT), Self-alignment Pretrained Bidirectional Encoder Representations from Transformers (SapBERT), cross-lingual knowledge-infused medical term embedding (CODER) and MedCPT. We describe them briefly in the follows:

#### ClinicalBERT Model

The ClinicalBERT [37] is an extension of Biomedical Bidirectional Encoder Representations from Transformers [38] trained further on discharge summary notes from the Medical Information Mart for Intensive Care III (MIMIC-III) database [39]. It was designed to handle the complexity and nuances of clinical text.

#### PubMedBERT Model

PubMedBERT [40] was specifically designed to capture domain-specific knowledge present in biomedical literature. It was initialized from Bidirectional Encoder Representations From Transformers (BERT) and trained further on the collection of PubMed abstracts.

#### SciBERT Model

SciBERT [41] is a BERT-based language model pretrained on 1.14 million full-text papers from Semantic Scholar. The corpus domain cuts across the field of computer science and the biomedical space.

#### SapBERT Model

SapBERT [42] is also a BERT-based model initialized from PubMedBERT. SapBERT was further pretrained on UMLS [28], which consists of a wide range of biomedical ontologies for >4 million concepts.

#### CODER Model

CODER [43] is another BERT-based model formulated to generate biomedical embeddings. It was also initialized from PubMedBERT. CODER was further pretrained using the concepts from the UMLS [28] and optimized to increase the embedding similarities between terms with the same concept unique identifier.

#### MedCPT Model

MedCPT [36] is a contrastive pretrained PubMedBERT-based model also formulated to generate biomedical text embeddings for multiple tasks.

#### Supervised Fine-Tuning

Given a set of patients’ notes (hereinafter also referred to as *notes*) as inputs and their corresponding diagnoses as outputs, we fine-tuned the pretrained models in a supervised fashion to classify diagnoses by feeding in a series of notes and their corresponding ground-truth diagnoses. Each note was a textual document describing a patient’s health condition and medical history. The ground-truth diagnosis of a note was the corresponding health condition of the patient. Multimedia Appendix 1 provides details of the models’ parameter settings. After fine-tuning, given a test set of notes, a model assigned probabilities to each ground-truth diagnosis for each note. The diagnosis with the highest probability corresponded to the model’s most confident prediction. We assigned ranks to each diagnosis in the order of their decreasing probability score for all our predictions. These ranks were further used to compute the mean reciprocal rank (MRR) for model evaluation (refer to the *Evaluation Metrics* section for details). We justify the use of ranking output probabilities across classes to compute the MRR because the probabilities generated by the classifier represent the classifier’s confidence in predicting each incidence. Supervised fine-tuning requires diagnosis-specific training data (availability of historic patient notes for each diagnosis) to deliver state-of-the-art performance. Unfortunately, labeled data are expensive to generate. This requirement makes it impractical to use a supervised fine-tuned model to diagnose those diseases without (many) notes for training. Hence, we used this method to make predictions only when training data were available.

#### Zero-Shot Learning

Given our focus on predicting diagnoses with few or 0 training samples, we included zero-shot learning methods as baselines. Leveraging the high quality of the aforementioned pretrained transformer embeddings, we adopted a zero-shot strategy by classifying patients’ notes based on their semantic similarity to potential diagnoses. This can be achieved by using pretrained models as biencoders [18,44]. Using this approach, we accounted for the diagnosis classes (classes without training samples) that the supervised fine-tuned models could not handle.

Given a patient’s note (our query) and the list of candidate diagnoses as labels, we used different variants of BERT as biencoders to encode queries and the full names of all ground-truth diagnoses to produce their respective representation vectors separately. Next, we computed their cosine similarity score and ranked each diagnosis for each query according to this score. The diagnosis with the highest cosine similarity became the model’s most confident diagnostic prediction (refer to Multimedia Appendix 1 for more details).

### Model Ensemble: Reciprocal Rank Fusion

The label retrieval process allowed the CliniqIR (retrieval-based model) to diagnose unseen conditions regardless of training data availability. This property is beneficial for diagnoses with little or no training data. On the other hand, a supervised fine-tuned model can draw much deeper insights from available historical case data. We adopted an ensemble strategy to combine the advantages of both paradigms.

In IR and general machine learning, ensemble strategies combine results from multiple models to produce a single joint output. Ideally, the ensemble model should produce a new output whose performance is superior to that of the individual constituent models. Several studies [32,45,46] have shown that high-performance gains can be achieved through model ensembling. One of the simplest ways to build such a model is to focus on applying a reranking heuristic to the ranks of each item in a model’s output list. Hence, we collected the ranked list of diagnoses from a CliniqIR model and that of the best-performing supervised fine-tuned model, ClinicalBERT, and combined the 2 lists. We then applied a modified version of the reciprocal rank fusion (RRF) [45] algorithm using equation 2 to merge their results and produce a single, final output list. Given a set *C*of concepts(diagnoses) to be ranked and a set of rankings *R*for all concepts obtained from each ensemble member (CliniqIR and ClinicalBERT), we computed the RRF score for each concept (*c*∈ *C*) as follows:



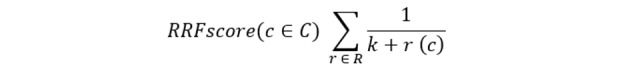

**(2)**


In the aforementioned equation, “*r* ∈ *R*” is the rank of concept *c*according to an ensemble member. We summed up the individual ranks of a concept from each ensemble member “*r*(*c*)” with *k* and computed the inverse. Previous work by Cormack et al [45] reported that setting *k*to 60 was the near-optimal choice for most of their experiments. Hence, we set *k* to 60 for all experiments. When concepts (diagnoses) had more than one training sample, we selected their individual ranks *r*from each ensemble member to compute the RRF score; otherwise, we selected ranks from the CliniqIR model. We used the RRF algorithm due to some key advantages: (1) it is a simple unsupervised method that eliminates the need for training samples, and (2) it effectively combines the results from various models without reliance on a weighting or voting mechanism.

### Experimental Setup

#### Data Sources

##### DC3 Data Set

The DC3 data set [30] was designed specifically for the evaluation of diagnostic support systems. The data set comprises 30 rare and difficult-to-diagnose cases compiled and solved by clinical experts in the *New England Journal of Medicine* Case Challenges. This data set lacks large, labeled training data, but it covers a wide range of diagnostic cases for various specialties. Therefore, we used this data set to determine the applicability of CliniqIR for diagnostic inference when the underlying patient condition is rare. Each case is a patient’s note and its corresponding true diagnosis written as free text. We mapped the true diagnoses to their UMLS concept IDs to produce test labels for evaluation consistency. When we did not find an exact matching term for a diagnosis, we considered the closest match returned by the UMLS browser. During the preprocessing step, we found that some cases in the DC3 data set had multiple terms representing a ground-truth diagnosis, making it difficult to find a single UMLS concept ID for such cases. To ensure an accurate mapping with the UMLS concept IDs, we split such cases into separate terms. For example, the case “Acute and chronic cholecystitis and extensive cholelithiasis with transmural gallbladder inflammation” was split into 2 separate terms: “Acute and chronic cholecystitis” and “Extensive cholelithiasis with transmural gallbladder inflammation.” Then, we mapped each case to its corresponding UMLS concept ID. Next, we computed the document frequency of all the true diagnoses (now represented as concepts) across all PubMed abstracts. In these cases, either of the concepts could be considered as the ground truth. As the data did not contain sufficient notes to train a model, we formulated this task as a zero-shot multiclassification problem. Specifically, we expected a model to predict the underlying condition given a patient’s note without labeled training data.

##### MIMIC-III Data Set

The MIMIC-III [39] is a freely accessible medical database that contains information on >50,000 intensive care unit patients. The data include laboratory events, vital sign measurements, clinical observations, notes, and diagnoses structured as *ICD-9-CM* (*International Classification of Diseases, Ninth Revision, Clinical Modification*), codes. We worked with the discharge notes for all experiments because they document a free-text synopsis of a patient’s hospital stay from admission to discharge. In MIMIC-III, each discharge note is mapped to multiple diagnoses ranked according to priority. We considered the highest-priority diagnosis to be the admission’s ground-truth diagnosis (and prediction target). We excluded admissions primarily for birth and pregnancy as they did not represent a primary pathological diagnosis. After preprocessing, the discharge notes contained 2634 unique *ICD-9-CM* diagnoses. We mapped these *ICD-9-CM* diagnoses to their corresponding UMLS concept IDs to calculate their TF across the knowledge resource (PubMed abstracts). The resulting unique diagnoses were associated with notes ranging from thousands of occurrences of frequent conditions, such as coronary atherosclerosis and aortic valve disorders, to rare ones, such as Evans syndrome and ehrlichiosis, with just a single instance forming a long-tailed distribution. A total of 902 diagnoses fell into the singleton category. One discharge note representing a specific diagnosis is insufficient to train and test a model. Thus, we reserved all diagnoses with only 1 available note for model testing. For diagnoses with <5 note samples, we reserved 1 sample for testing, and the rest were included in model training. We split the remainder of the data set (instances of diagnoses with ≥5 associated notes) into training, validation, and testing sets in the ratio 70:15:15; this split resulted in the training set containing notes representing 1732 unique diagnoses and the test set containing notes representing a total of 2634 unique diagnoses (refer to Multimedia Appendix 1 for more details). For models that did not require training (eg, the retrieval model), we used the validation and training sets for hyperparameter tuning purposes and the test set for final model evaluations.

#### Baselines

Previous studies and competitions, such as the TREC clinical decision support track, have emphasized the development and evaluation of IR systems to aid clinical decision-making. While these systems are commonly used for evidence-based literature searches, our study explored their adaptation for direct literature-guided diagnosis prediction. Although a direct comparison to the systems in the TREC clinical decision support track was not possible, insights gained from these competitions informed the engineering of our retrieval system. To evaluate our model, we used 2 transfer learning techniques—supervised fine-tuning and zero-shot classification methods (refer to the *Pretrained Transformer Models* section)—because of their performance in scenarios where labeled data are limited or unavailable. In addition, some studies [47-49] have shown pretrained language models to attain superior performance to that of count vector–based models and traditional supervised methods in various medical tasks. We used “Z” to identify when models were used in a zero-shot classification setting, an “S” for supervised fine-tuning, and “CliniqIR” when models were used in a retrieval setting. Table 1 provides details.

**Table 1:** Overview of the experiments conducted using the different models and their task description.

Experiment	Models used	Task description
Retrieval-based experiments (CliniqIR)	BM25^a^ and MedCPT^b^	Models retrieved relevant abstracts to inform diagnostic predictions.
Zero-shot experiments (Z)	ClinicalBERT^c^, PubMedBERT^d^, CODER^e^, SapBERT^f^, and MedCPT	Models classified diseases in a zero-shot setting without previous task-specific training.
Supervised experiments (S)	ClinicalBERT	Models were fine-tuned using labeled data for enhanced disease prediction accuracy.

^a^BM25: Best Match 25.

^b^MedCPT: Medical Contrastive Pretrained Transformers.

^c^ClinicalBERT: Clinical Bidirectional Encoder Representations from Transformers.

^d^PubMedBERT: PubMed Bidirectional Encoder Representations from Transformers.

^e^CODER: cross-lingual knowledge-infused medical term embedding.

^f^SapBERT: Self-alignment Pretrained Bidirectional Encoder Representations from Transformers.

#### Evaluation Metrics

In our experiments, each model returned a ranked list of diagnoses analogous to a ranked list of differential diagnoses formulated by a medical expert. Given a query and a list of ranked items produced by a model, a simple classification accuracy metric tracks whether the model made the correct prediction at the top of the list. Instead, we used the MRR [50] because it told us *where* the true diagnosis was placed in the list in equation 3. If a model returned the reference diagnosis at rank 1 (ie, at the top of the list), the reciprocal rank (RR) was 1; if the most appropriate item was at rank 2, then the RR was 0.5. The RR decreases as the relevant item moves farther down the list. We calculated the MRR by computing the average RR across admissions. An MRR of 1 meant that the model returned the correct diagnosis at the top of its list for every patient, and an MRR of 0 implied that the model never produced a correct diagnosis. Mathematically, the MRR can be represented as follows:



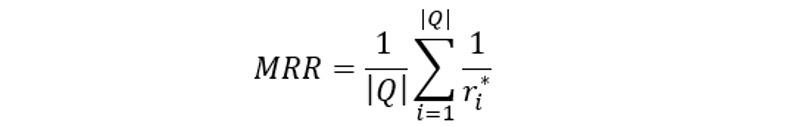

**(3)**


where *|Q|* denotes the total number of queries and denotes the rank of the correct diagnosis. We also calculated the mean average precision (MAP) to evaluate the balance between precision and recall of the retrieval systems (for details, refer to Multimedia Appendix 1).

### Ethical Considerations

No ethics approval was pursued for this research, given that the data were publicly accessible and deidentified.This aligns with the guidelines outlined by the US Department of Health and Human Services, Office for Human Research Protections, §46.101 (b)(4) [51].

## Results

### CliniqIR Models Retrieved Useful Literature and Meaningful Concepts

Table 2 showcases qualitative results for 3 selected queries, displaying the top 3 documents retrieved by the CliniqIR model. Notably, the retrieved articles and their corresponding concepts demonstrated clear relevance to the ground-truth diagnoses of the respective queries.

**Table 2:** Qualitative overview of the top documents and concepts retrieved for 3 selected queries along with their respective correct concepts. This table illustrates the types of results our system generates for each query, showing the alignment with the ground-truth concepts.

Ground-truth diagnosis, and top 3 documents			Retrieved concepts
**1. Viral pneumonia**			
	Relevant article 1—PMID^a^ 15336585: Cases from the Osler Medical Service at Johns Hopkins University. Diagnosis: P. carinii pneumonia and primary pulmonary sporotrichosis	{“C1956415”: [“paroxysmal nocturnal dyspnea”], “C0239295”: [“esophageal candidiasis”], “C0236053”: [“mucosal ulcers”], “C1535939”: [“Pneumocystis”], “C0031256”: [“petechiae”], “C0006849”: [“thrush”], “C0011168”: [“dysphagia”]}	
	Relevant article 2—PMID 32788269: A 16-Year-Old Boy with Cough and Fever in the Era of COVID-19	{“C0746102”: [“chronic lung disease”], “C0004096”: [“asthma”], “C0009443”: [“cold”], “C0206750”: [“Coronavirus”], “C0018609”: [“h disease”]}	
	Relevant article 3—PMID 30225154: Meningococcal Pneumonia in a Young Healthy Male	{“C3714636”: [“pneumonias”], C1535950”: [“GI inflammation”]}	
**2. Hypoparathyroidism**			
	Relevant article 1—PMID 34765380: A Challenging Case of Persisting Hypokalemia Secondary to Gitelman Syndrome	{“C0220983”: [“metabolic alkalosis”], “C0151723”: [“hypomagnesemia”], “C0020599”: [“hypocalciuria”], “C0014335”: [“enteritis”], “C0012634”: [“Diagnosis”], “C0235394”: [“wasting”], “C0271728”: [“Hyperreninemic hyperaldosteronism”], “C0268450”: [“gitelman syndrome”], “C3552462”: [“Tubulopathy”]}	
	Relevant article 2—PMID 27190662: Suppression of Parathyroid Hormone in a Patient with Severe Magnesium Depletion	{“C0151723”: [“hypomagnesemia”], “C0030554”: [“paresthesias”], “C0020598”: [“hypocalcemias”], “C0020626”: [“Low parathyroid hormone”], “C0030517”: [“Parathyroid”], “C0033806”: [“pseudo hypoparathyroidism”]}	
	Relevant article 3—PMID 28163524: Afebrile Seizures as Initial Symptom of Hypocalcemia Secondary to Hypoparathyroidism	{“C0020626”: [“Hypoparathyroidism”], “C0012236”: [“DiGeorge syndrome”], “C0863106”: [“afebrile seizures”], “C0030353”: [“papilledema”], “C0020598”: [“Hypocalcemias”], “C0012634”: [“Diagnosis”], “C0042870”: [“Vitamin D deficiency”]}	
**3. Intracerebral hemorrhage**			
	Relevant article 1—PMID 9125737: A 36-year-old woman with acute onset left hemiplegia and anosognosia	{“C0020564”: [“enlargement”], “C0019080”: [“hemorrhage”]}	
	Relevant article 2—PMID 25830084: Multiple extra-ischemic hemorrhages following intravenous thrombolysis in a patient with Trousseau syndrome: case study.	{“C2937358”: [“Intracerebral hemorrhage”], “C0151699”: [“intracranial hemorrhage”], “C0019080”: [“hemorrhages”], “C0020564”: [“enlargement”], “C0021308”: [“infarct”], “C0022116”: [“ischemia”]}	
	Relevant article 3—PMID 1434057: A case of recurrent cerebral hemorrhage considered to be cerebral amyloid angiopathy by cerebrospinal fluid examination.	{“C0472376”: [“thalamic hemorrhage”], “C2937358”: [“cerebral hemorrhage”], “C0019080”: [“bleeding”], “C0023182”: [“cerebrospinal fluid leak”]}	

^a^PMID: PubMed ID.

### CliniqIR Models Yielded State-of-the-Art Performance for Rare and Complex Diagnoses

We examined the retrieval-based models’ (CliniqIR) performance on the DC3 data set to show their applicability for rare and complex diagnostic cases. The absence of training data for this data set implied that supervised learning would not be applicable and the models could only make predictions in an unsupervised or zero-shot setting. Hence, on this data set, we compared the CliniqIR models’ performance to that of pretrained transformers in a zero-shot setting. In contrast to the CliniqIR model, which creates its own set of labels, we supplied the pretrained transformers with a range of potential diagnoses for each query to enable zero-shot predictions. This gave the models a significant advantage over their use in a real-world setting, where such information would not be readily available. Table 3 shows the MRR of the chosen models on the DC3 data set. Even with the supporting assumption that the range of possible diagnoses was known to the pretrained models, the CliniqIR models outperformed them with an MRR of 0.35 and 0.32 for CliniqIR_BM25 and CliniqIR_MedCPT, respectively. This means that, on average, CliniqIR_BM25 and CliniqIR_MedCPT were more likely to return the correct diagnosis within the top 3 predictions for a case.

The MRR scores of the pretrained zero-shot methods were similar to one another but markedly lower; the scores were 0.15, 0.22, 0.25, 0.25, 0.24, and 0.18 for ClinicalBERT, PubMedBERT, SciBERT, CODER, SapBERT, and MedCPT, respectively. Our results show that the CliniqIR models are capable of making useful predictions in the case of rare and complex diagnoses with limited or no training data availability.

**Table 3:** Performance evaluation of the models on the DC3 data sets across all case. The retrieval-based models, denoted using “CliniqIR” gave the best overall performance compared to the zero-shot models, denoted using “Z.”

Model used	Mean reciprocal rank
ClinicalBERT^a^ (Z)	0.15
PubMedMERT^b^ (Z)	0.22
SciBERT^c^ (Z)	0.25
CODER^d^ (Z)	0.25
SapBERT^e^ (Z)	0.24
CliniqIR_BM25	*0.35* ^f^
MedCPT^g^ (Z)	0.18
CliniqIR_MedCPT	*0.32*

^a^ClinicalBERT: Clinical Bidirectional Encoder Representations from Transformers.

^b^PubMedBERT: PubMed Bidirectional Encoder Representations from Transformers.

^c^SciBERT: Scientific Bidirectional Encoder Representations from Transformers.

^d^CODER: cross-lingual knowledge-infused medical term embedding.

^e^SapBERT: Self-alignment Pretrained Bidirectional Encoder Representations from Transformers.

^f^Highest mean reciprocal rank is italicized.

^g^MedCPT: Medical Contrastive Pre-trained Transformers.

### Performance on MIMIC-III

#### Supervised Prediction Models Failed at Making Rare Diagnoses

When training data are available, supervised models are preferred. Thus, we investigated the effectiveness of a supervised learning approach for a highly imbalanced data set such as MIMIC-III. We fine-tuned the pretrained models to predict diagnoses using available clinical notes. Diagnoses were categorized based on the frequency of associated notes to show how training data availability affects a supervised model’s predictive capacity. A total of 902 diagnoses had no training data (only 1 note representative in MIMIC-III), whereas 1732 had at least one training sample (≥2 note representatives in MIMIC-III). Predictions were made only for the 1732 diagnoses, excluding those without training data. We introduced sample weights in the loss function to handle the enormous data imbalance. This approach weighs the loss computed for samples differently depending on their class training size. Our results in Figure S2 in Multimedia Appendix 1 show that ClinicalBERT performed best among all pretrained models. Hence, we used ClinicalBERT as our supervised baseline for the remainder of our experiments.

After training ClincalBERT, we tested it on different clinical note frequency–based categories (Table 4). In Table 4, we observed that the MRR score of the ClinicalBERT model was higher for diagnosis categories with many training examples (>10 notes). In addition, in the data set category with 1 to 10 notes available per diagnosis (1<notes≤10), ClinicalBERT obtained a low MRR score of 0.07. An MRR of 0.08 indicates that, on average, ClinicalBERT returned the correct diagnosis for a case among its top 13 predictions for these diagnoses. While the model could not perform predictions for 902 diagnoses due to the lack of training data, the drastic decline in ClinicalBERT’s performance also indicates that the model is not suitable for making predictions for diagnoses with <10 clinical notes available for training. We also noticed a decline in performance for diagnoses with training samples between 500 and 750. This was likely due to many diagnoses having similar symptoms and manifestations. Therefore, the supervised learning approaches struggle to find a fine delineation of boundaries between similar classes without sufficient training data.

**Table 4:** Performance of the best-performing fine-tuned supervised model, Clinical Bidirectional Encoder Representations from Transformers on the Medical Information Mart for Intensive Care III data set. We categorized the results by the frequency of training note representation per diagnosis.

Data set category	Mean reciprocal rank
0 note	—^a^
1≤Notes≤10	0.08
10<Notes≤50	0.33
50<Notes≤100	0.49
100<Notes≤250	0.52
250<Notes≤500	0.57
500<Notes≤750	0.44
750<Notes	0.41
0<Notes	0.37

^a^Not applicable.

#### CliniqIR Models Outperformed ClinicalBERT for Rare Diagnoses

**Figure 4:**
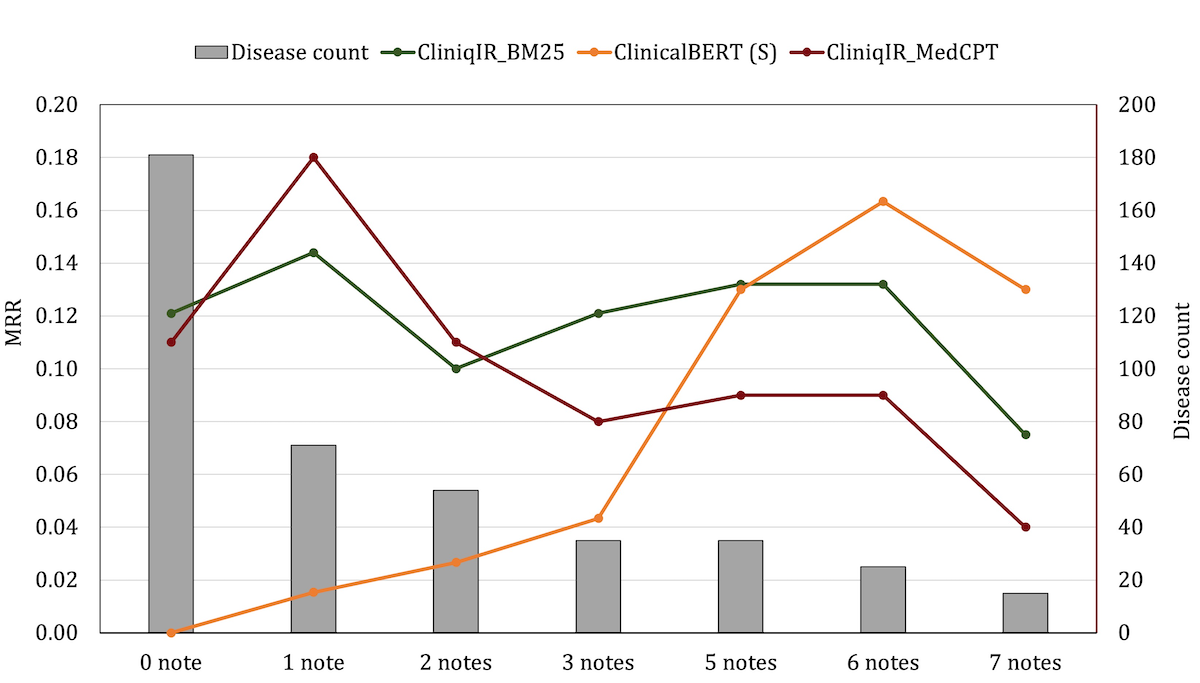
Mean reciprocal rank (MRR) results for CliniqIR-based models and Clinical Bidirectional Encoder Representations from Transformers (ClinicalBERT) when predicting diagnoses with training sample sizes of 0, 1, 2, 3, 5, 6, and 7. Results indicate that the CliniqIR-based models perform best when the training sample size is between 0 and 5. However, ClinicalBERT performs best as training data size increases. “S” denotes that the ClinicalBERT model was used in a supervised setting.

The objective of this experiment was to determine to what extent the CliniqIR models can be used in place of a supervised model when the training sample size is small. Results in Figure 4 show that CliniqIR-based models performed better than ClinicalBERT for diagnoses with up to 3 training samples. In addition, CliniqIR_BM25 and ClinicalBERT had similar MRR scores for diagnoses with 5 training samples. The average MRR score for the CliniqIR-based models was approximately 0.1 across most categories except for diagnoses with at least 7 training samples. This result indicates that, on average, their correct prediction for a query was ranked 10th on the list. The disease count bars in Figure 4 (in gray) also show that the number of diseases with <5 training samples was more than twice the number of diseases with >5 training samples. Thus, CliniqIR allows for more disease coverage and also generalizes well for cases with low note availability. This result confirms that, while supervised models may perform well with sufficient labeled training data, CliniqIR-based models’ performance stands out as remarkable for diagnoses in the low-data regime.

#### CliniqIR Models Outperformed Zero-Shot Baselines for Rare Diagnoses

**Figure 5:**
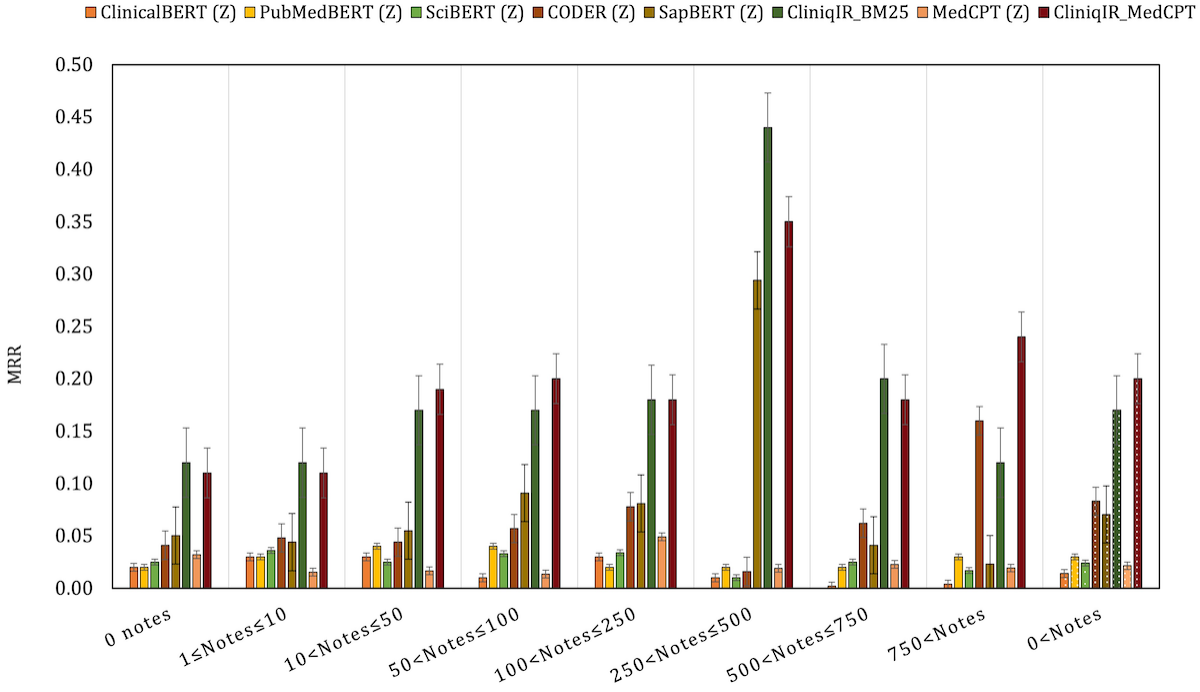
Performance evaluation of CliniqIR models and each pretrained zero-shot baseline on the Medical Information Mart for Intensive Care III data set. We categorized the results by the frequency of note representative per diagnosis. “Z” represents models used in a zero-shot setting. The CliniqIR models performed best across data set categories in the low-resource regime. ClinicalBERT: Clinical Bidirectional Encoder Representations from Transformers; CODER: cross-lingual knowledge-infused medical term embeddin; MedCPT: Medical Contrastive Pre-trained Transformers; MRR: mean reciprocal rank; PubMedBERT: PubMed Bidirectional Encoder Representations from Transformers; SapBERT: Self-alignment Pretrained Bidirectional Encoder Representations from Transformers; SciBERT: Scientific Bidirectional Encoder Representations from Transformers.

To further demonstrate the utility of CliniqIR models for diagnoses with little or no training samples, we compared its performance to that of the pretrained models in a zero-shot setting. As mentioned previously, the MIMIC-III data set comprises >2634 diagnoses, but the supervised fine-tuned models were effective only for a subset of diagnoses with training data; 902 diagnoses had no training samples at all. In zero-shot settings, pretrained models can make predictions without reliance on training data. In Figure 5, we observe that CliniqIR models outperformed the zero-shot pretrained models across most data set categories, especially when diagnoses had a low number of associated training notes. The highest and lowest MRR scores obtained by CliniqIR_BM25 were 0.44 and 0.12, respectively, whereas CliniqIR_MedCPT’s highest and lowest scores were 0.35 and 0.11.

Among the zero-shot baseline, CODER and SapBERT’s performances were better across most data set categories. However, in the category in which all diagnoses were considered (diagnoses with >0 notes), CODER outperformed SapBERT, obtaining a maximum and minimum MRR score of 0.16 and 0.02, respectively. These MRR scores indicate that, on average, both CliniqIR models returned the correct diagnosis for a case among their top 5 predictions. In contrast, the best-performing pretrained zero-shot baselines, CODER and SapBERT, returned an accurate diagnosis for a query among their top 15 and 12 predictions, respectively.

### Ensemble Models Yielded State-of-the-Art Performance

We have shown that CliniqIR models deliver valuable diagnostic decision support in the setting of limited or unavailable training data. On the other hand, a supervised pretrained model such as ClinicalBERT is an efficient alternative when training data are abundant. To combine the strengths of both models, we used the RRF algorithm as an ensemble strategy. The RRF algorithm combines the ranks of all the ensemble members (a CliniqIR model and a supervised model) to produce a new ranked list of diagnoses for a given patient’s clinical note. We hypothesized that creating an ensemble with both models would boost predictive performance across various diagnoses regardless of the availability of associated clinical notes.

**Figure 6:**
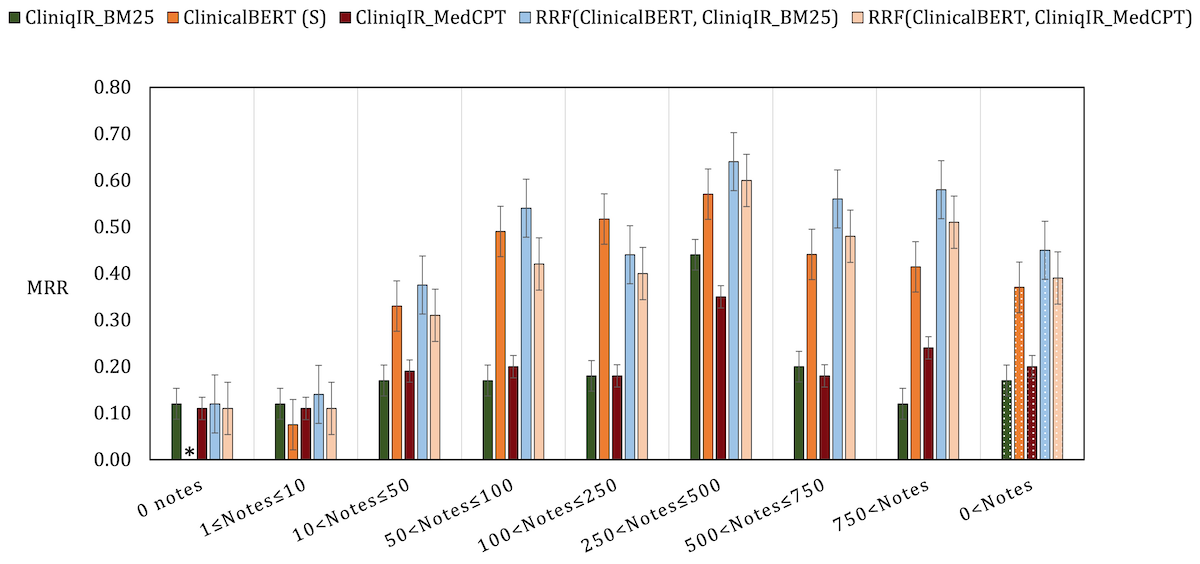
Performance evaluation of the models on the Medical Information Mart for Intensive Care III data set before and after the ensemble. Adopting the reciprocal rank fusion (RRF) algorithm as an ensemble strategy boosted predictive performance across the data set. The Clinical Bidirectional Encoder Representations from Transformer (ClinicalBERT) model cannot directly make predictions for diagnoses with no training samples. Hence, we used “*” to mark such data set categories. The letter “S” denotes that ClinicalBERT was used as a supervised model. MedCPT: Medical Contrastive Pre-trained Transformers; MRR: mean reciprocal rank.

To implement the RRF algorithm introduced in the *Model Ensemble: Reciprocal Rank Fusion* section, we used ClinicalBERT and a CliniqIR model to obtain separate ranked lists for each diagnosis and concept across queries. We compared the predictive performance of each individual model to that of the ensemble in terms of MRR for each note availability category. Figure 6 shows the output of the experiments before and after fusing the predicted ranks from both models.

Interestingly, for either of the CliniqIR models used, the ensemble model improved the overall average performance for predicting a wide range of diagnoses (>0 notes) in the MIMIC-III data set. We also found that the RRF ensemble successfully boosted performance across diagnosis categories with both high and low note availability. On average, the RRF ensemble model performed better than either of its constituent models. Notable exceptions include the categories in which the individual mean average precision of both CliniqIR_BM25 and CliniqIR_MedCPT was <0.50 (refer to Multimedia Appendix 1 for details) and in the 100 to 250 training example range, in which the ensemble was slightly worse than the supervised model. In all other conditions, the interaction between both models (the ensemble) led to better performance.

## Discussion

### Principal Findings

With thousands of known diseases potentially causing a patient’s condition, it is often difficult—even for experienced clinicians—to accurately diagnose every disease. Unlike the pretrained models that require user input of possible diagnoses before predictions can be made, CliniqIR represents a potential decision support tool that takes advantage of the wealth of medical literature in PubMed to generate a differential diagnosis. Our study evaluated CliniqIR’s ability to formulate differentials and predict uncommon diagnoses with few or no training examples, reflecting conditions easily missed in real-life practice. Results comparing CliniqIR’s performance to those of pretrained biomedical transformers in supervised and zero-shot settings highlight CliniqIR’s ability to operate successfully as an unsupervised model. Therefore, our model’s strength is not limited to rare and infrequent diagnosis prediction, and our model is also a useful tool for generating a first-stage differential diagnosis list. As such, a diagnostic decision support tool such as CliniqIR can enhance physician differential diagnoses and facilitate more efficient diagnoses by providing literature-guided suggestions. Beyond disease prediction, CliniqIR also demonstrates relevance in medical education as a patient-centric literature search tool. Our study demonstrated its ability to accurately cultivate a list of PubMed literature relevant to a patient’s clinical narrative. This functionality could greatly improve physician researcher efficiency in performing dedicated literature reviews on behalf of their patients.

In the era of large complex neural models, it is critically important that diagnostic support tools remain simple and interpretable. In health care, where decision-making is critical and patient outcomes are at stake, clinicians’ ability to understand and trust the inner workings of a diagnostic tool is paramount. In response, CliniqIR is built on retrieval systems that use simple and transparent weighting schemes to retrieve and rank important terms in a collection of documents. This transparency fosters trust in the tool’s accuracy and facilitates collaboration between the tool and the health care professionals, leading to ongoing model refinement as well as enhanced clinical decision-making.

### Limitations

The medical field is witnessing a growing trend in applications built on generative large language models [52]. While our work used a simpler approach, it remains valuable in scenarios with limited access to significant computing resources. In addition, it serves as a proof of concept for a retrieval-augmented medical model, potentially leading to enhanced explainability and accuracy for large language models in the health care domain.

Our study has 3 potential limitations. First, CliniqIR’s knowledge source is limited to abstracts in PubMed, which has well-known publication biases toward certain diagnoses [53,54]. Therefore, the use of a single knowledge resource limits CliniqIR’s generalizability to diseases and conditions not represented in the PubMed corpus. For instance, conditions such as COVID-19 and Alzheimer disease or rare diseases such as sarcoidosis and cholangitis are covered in thousands of published literature entries, whereas other conditions such as “cellulitis and abscess of the leg” or “closed fracture of the sternum” may receive less attention. Future studies will involve a review of seemingly unrepresented diagnostic codes by linking them back to their parent diagnostic codes to ensure appropriate mapping between diagnosis codes and PubMed.

Second, our main experimental results were restricted to diagnoses with at least 100 PubMed abstract representatives. We identified a significant number of *ICD-9-CM* codes in MIMIC-III with no associated medical literature among the 33 million PubMed abstracts (an overview of MIMIC-III diagnosis distribution classes can be found in Multimedia Appendix 1). We also found that CliniqIR’s predictive performance improved with increasing PubMed coverage of the diagnosis, guiding our decision to establish the 100-abstract inclusion criterion for diagnoses (Multimedia Appendix 1). Future work will combine information from biomedical journals, medical textbooks, and Wikipedia for wider disease coverage.

Third, our MIMIC-III experiments limited the input to patient discharge summaries containing a succinct synopsis of a patient’s hospital stay, including symptoms, diagnostic evaluation, clinical progression, and treatment information. In real-world clinical situations, such complete retrospective information would not be available during the initial diagnostic process. Therefore, the results presented in this paper represent a first feasibility study of CliniqIR and highlight some of the difficulties involved in developing diagnostic support tools.

### Conclusions

In this study, we presented CliniqIR, an unsupervised retrieval-based model that leverages unstructured knowledge resources to aid in the diagnostic process. We showed that the CliniqIR models outperformed a supervised fine-tuned pretrained clinical transformer model in predicting diagnoses with <5 training samples. We also demonstrated that CliniqIR outperformed pretrained clinical transformers in making predictions for rare and complex conditions in a zero-shot setting. While many existing research studies on diagnostic prediction have focused on one disease at a time or only on highly prevalent conditions, we combined the strengths of CliniqIR and supervised learning to build a single ensemble model that aids in diagnosing a broad spectrum of conditions regardless of training data availability. Overall, our study reveals the potential of IR-based models in aiding diagnostic decision-making in an efficient, transparent, and educational manner. This work will direct future studies to facilitate successful application of machine learning and IR to building robust and accurate clinical diagnostic decision support tools.

## Appendix

Multimedia Appendix 1 Details on model implementation, configuration, and performance comparisons across various data sets and configurations.

## Abbreviations

BERT: Bidirectional Encoder Representations From Transformers
ClinicalBERT: Clinical Bidirectional Encoder Representations from Transformers
CODER: cross-lingual knowledge-infused medical term embedding
DDSS: diagnostic decision support system
ICD-9-CM: International Classification of Diseases, Ninth Revision, Clinical Modification
IR: information retrieval
MAP: mean average precision
MedCPT: Medical Contrastive Pre-trained Transformers
MIMIC-III: Medical Information Mart for Intensive Care III
MRR: mean reciprocal rank
PubMedBERT: PubMed Bidirectional Encoder Representations from Transformers
RR: reciprocal rank
RRF: reciprocal rank fusion
SapBERT: Self-alignment Pretrained Bidirectional Encoder Representations from Transformers
SciBERT: Scientific Bidirectional Encoder Representations from Transformers
TF: term frequency
TF-IDF: term frequency–inverse document frequency
TREC: text retrieval conference
UMLS: Unified Medical Language System

## Data Availability

The datasets analyzed during this study are available. The Medical Information Mart for Intensive Care III data set can be accessed with permission via the work by Johnson et al [39]. The DC3 data set can be accessed via the work by Eickhoff et al [30]. The code to implement this work is also available [55].
