# Supplementary material for: Retrieval-Based Diagnostic Decision Support: Mixed Methods Study
Source: JMIR Med Inform. 2024 Jun 19;12:e50209. doi: 10.2196/50209 (PMC11222760; doi:10.2196/50209)
Supplement: Multimedia Appendix 1 [file medinform_v12i1e50209_app1.docx]

**Multimedia Appendix I**

# S1 Retrieval system experimental details.

The MIMIC-III datasets contained queries (patient notes) with an average length of 1472 words, while the DC3 queries were much shorter While the MedCPT retrieval model limited input to 512 tokens, the BM25 Lucene model offered a higher default limit of 1024 words. However, processing longer queries introduces a trade-off between retrieval speed and capturing relevant information. To address this, we tested various query lengths for BM25 and MedCPT to find a balance between runtime efficiency and retaining essential query details. We tested different maximum input lengths for MedCPT, finding that the default setting of 64 tokens achieved the best Mean Reciprocal Rank (MRR). For BM25 queries, we pre-processed the data using QuickUMLS. To explore the impact of query length, we randomly selected 300 queries from the MIMIC-III validation set and used increasing lengths (from 100 to 1000 words with increments of 100) as input. Figure S1 demonstrates that while total runtime increases with query length (as expected), MRR initially shows instability with very short queries. However, it stabilizes around 700 words and then declines slightly at 1000 words. Based on this, we chose a query length of 700 words for all subsequent experiments. This choice aligns with the observation that patient summaries usually have the history of present illness within the first few hundred words, and our findings suggest that 700 words strike a good balance between response time and capturing relevant information.


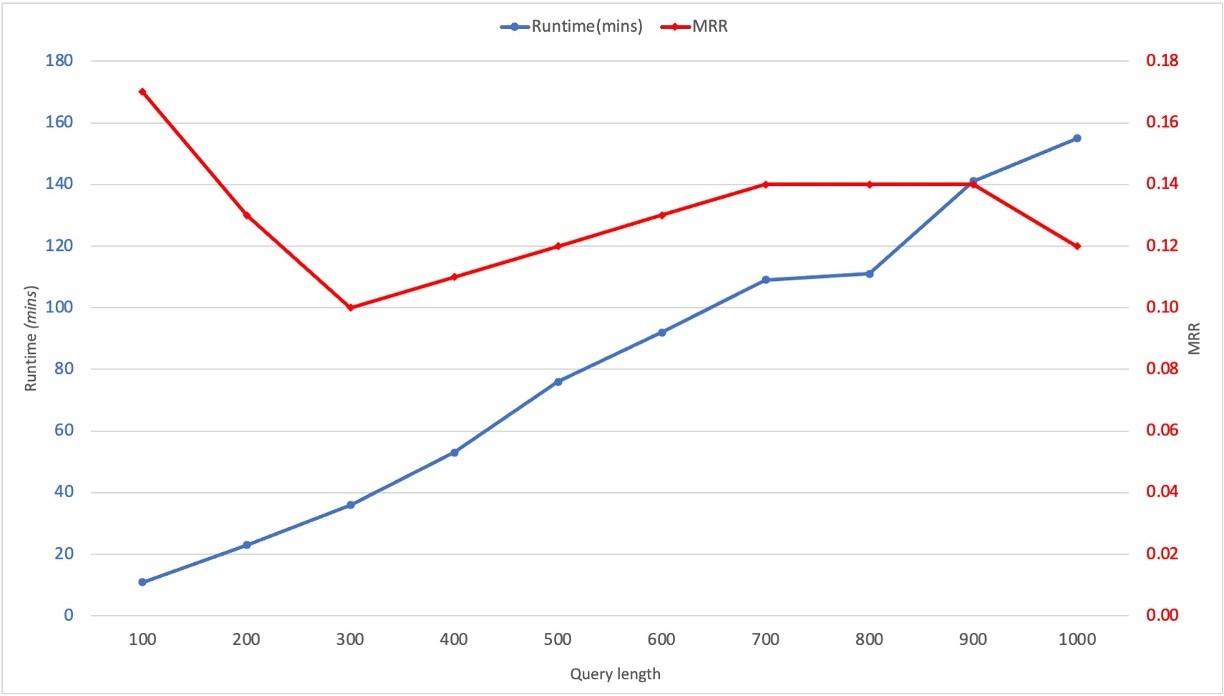


Figure S1: CliniqIR_BM25’s MRR and runtime (recorded in minutes) performance comparison of MIMIC-III notes with varying query lengths for 300 queries. The figure shows that the model’s runtime increases as we increase the input query length.

# S2 Parameter setup for the supervised fine-tuned models

To fine-tune the pre-trained models for classifying diagnoses, a series of queries (patient notes) and their ground truth diagnoses (concepts) are fed to the model. After a series of hyper-parameter tuning, we selected the best-performing parameters and used the same settings for all models. Random seeds of 42, 10, and 1 were used for all experiments, and training was performed over 20 epochs with a batch size of 8. The maximum number of token embeddings to be processed at a time was 512. We adopted the Adam optimizer with a learning rate of 1e-05 and a dropout rate of 0.1 for all models. After training, given a test set of queries, probabilities are assigned to each ground truth diagnosis for each query. The diagnosis with the highest probability is selected as the model’s most confident prediction. Ranks are assigned to each diagnosis (concept) according to their decreasing probability score for all our experiments. To handle class imbalance, we used a weighted cross-entropy loss function. We calculated each concept’s weight using Equation 4. Figure S2 represents the result of finetuning the models on the MIMIC-III datasets. In (Figure S2), we observed that the performance (MRR score) of the supervised models is higher for diagnoses categories with many training examples (*>* 10). However, ClinicalBERT performed best on average. Thus, we use it as our main supervised method for all other experiments.

|  | ${Weight}_{c}=\frac{1}{{Sample size of minimum class * Sample size of class}_{c}}$ | (Equation 4) |
| --- | --- | --- |


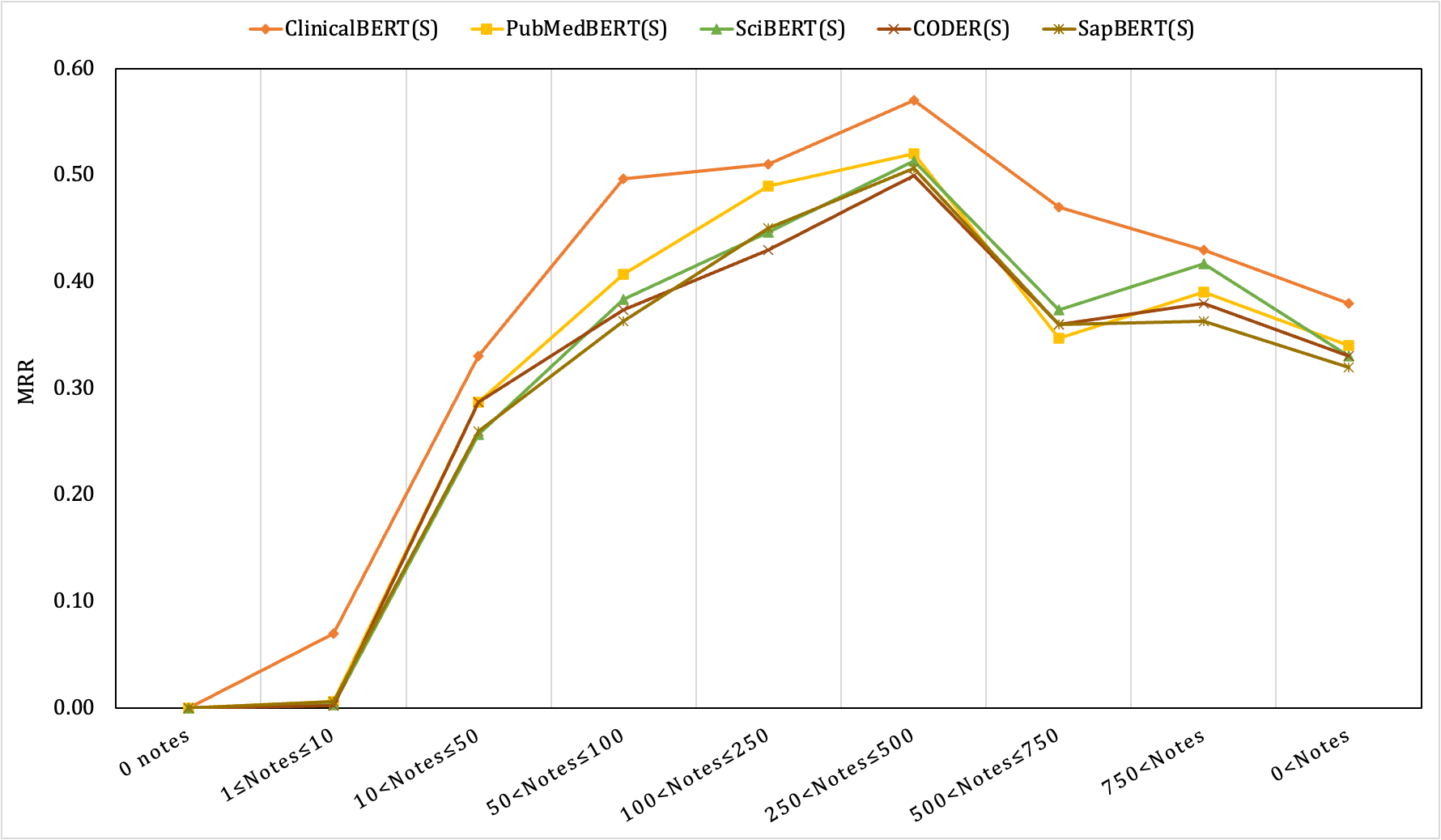


Figure S2: Performance evaluation of the fine-tuned pre-trained models across different dataset categories on the MIMIC-III datasets. We categorized the results by the frequency of note representative per diagnosis. The ClinicalBERT model obtains a high MRR score across most dataset categories. Note that via supervised fine-tuning, the models cannot make direct predictions for diagnoses without training samples. Hence, they are unable to make predictions in such dataset categories. We use the letter ‘S’ to denote that the models were used in a supervised setting.

# S3 Bi-encoders as zero-shot baselines

The use of pre-trained models as Bi-Encoders is a popular choice when we need to compare words or sentences in a vector space. We can efficiently compare words or sentences by independently passing them into a Bi-Encoder and computing the cosine similarity of their output embeddings. Since prior training is not required for decision-making, this method can be considered a zero-shot learning approach. We used a similar strategy to make predictions for diagnoses without relying on their training examples. Using this approach, we accounted for the diagnosis’s classes (classes without training samples) that the supervised fine-tuned could not handle. Hence, we used various pre-trained transformers as Bi-encoders for zero-shot performance baselines.

Given a patient’s note (query) and the list of candidate diagnoses as labels, as shown in (Figure S3), we used Bi-encoders first to produce their respective representation vectors separately. We used different variants of BERT as bi-encoders to encode queries and the full names of all ground truth diagnoses. Next, we computed the cosine similarity score between the embedding vectors of each query and the ground truth diagnoses. Finally, we ranked each diagnosis for each query according to the cosine similarity score. The diagnosis with the highest cosine similarity becomes the model’s most confident diagnostic prediction.


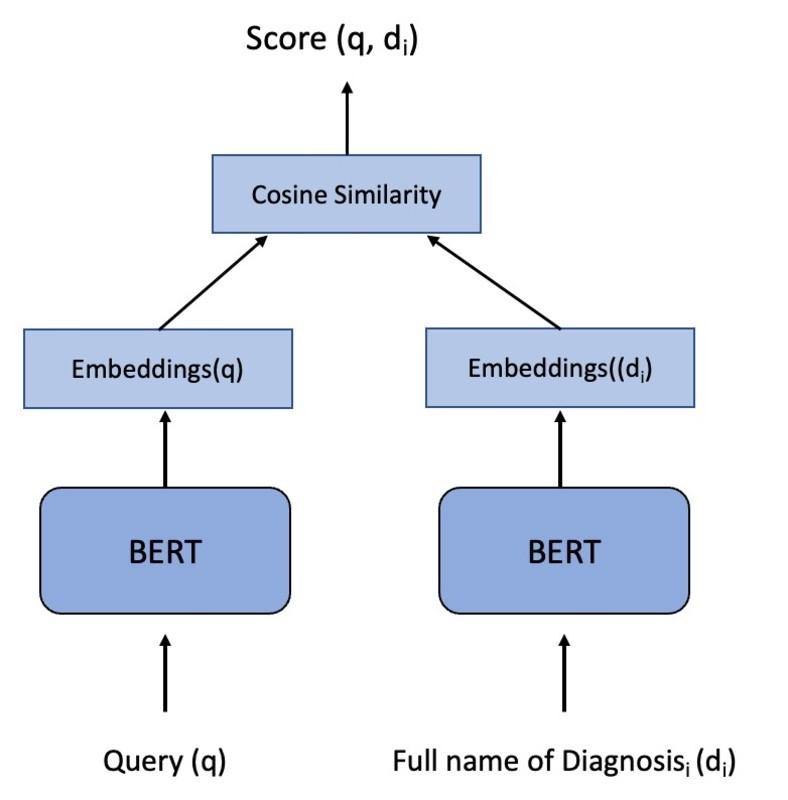


Figure S3: A Bi-encoder architecture illustrating the implementation of the BERT-based zero shot baselines. Bi encoders produced for a given input text a contextual embedding. We passed a query (patient’s note) and the full name of a diagnosis independently to a BERT-based model, which results in their embeddings q and *d_i_*. These sentence embeddings can then be compared using cosine similarity.

# S4 Distribution of MIMIC-III discharge summary notes across diagnoses

We filtered out notes mapped to diagnoses that are birth or pregnancy-related. After pre-processing, the discharge notes contained 2,634 unique diagnoses. The unique diagnoses range from thousands of occurrences for frequent conditions to rare ones, with just a single instance forming a long-tail distribution see (Figure S4). Nine hundred and two diagnoses (902) fall into the singleton category. One discharge note representative for a specific diagnosis is insufficient to train and test a model. Thus, we reserved all diagnoses with only one available note for model testing. For diagnoses with less than five note samples, we reserved one sample for testing purposes and the rest for model training. We split the remainder of the dataset (instances of diagnoses with five or more associated notes) into training, validation, and testing sets in the ratio 70:15:15. This means that diagnoses with less than 20 associated notes will have at most 14 samples available for model training purposes which is significantly less than diagnoses with thousands of note sample.


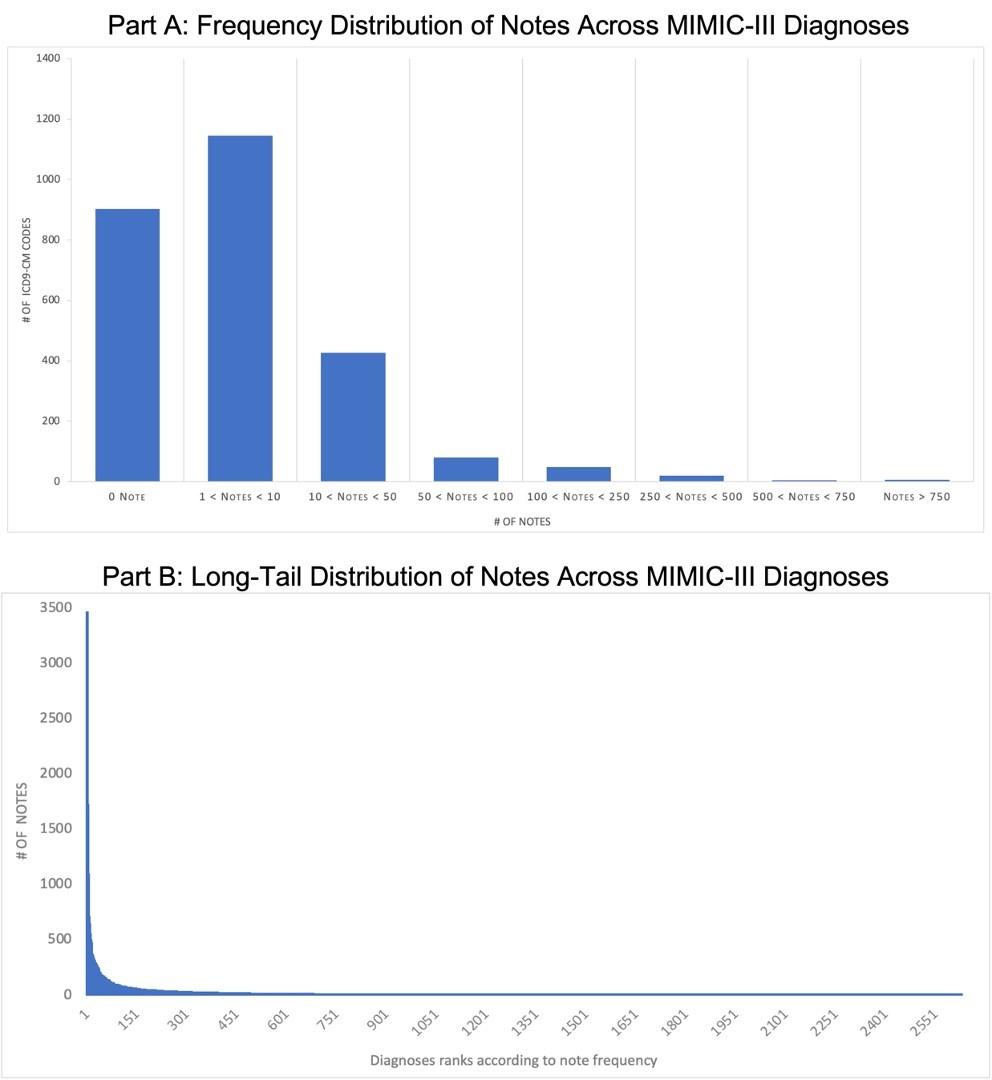
 Figure S4: Distribution of notes available for MIMIC-III diagnoses. *Part A:* The first bar to the right indicates that 902 diagnoses had only one corresponding admission. The figure shows that the number of diagnoses with low observation frequencies surpasses that with high frequencies. *Part B:* The figure shows a long-tail distribution in the number of admissions representatives for MIMIC-III diagnoses. The long-tail distribution attributes of MIMIC-III show that some diagnoses will have many training samples, while others will have few or no training samples.

# S5 Distribution of diagnoses categories across PubMed abstracts

We computed the PubMed document frequency of all the unique concept IDs (ground truth diagnoses) in the MIMIC-III and DC3 datasets. We observed a vast disparity in the document frequency across the MIMIC-III concept IDs. Over 1674 concept IDs had zero document frequency across the 33 million PubMed abstracts. In addition, 214 concept IDs had document frequencies less than or equal to ten, and only 571 concept IDs had document frequencies greater than or equal to one hundred. Since our retrieval models rely on PubMed abstracts as their source of knowledge, in all our major experiments, CliniqIR and the pretrained models were used to make predictions for diagnoses with at least a hundred document representatives across the 33 million PubMed abstracts. 571 unique diagnoses fell under the category of concepts with at least one hundred PubMed mentions. The 571 diagnoses covered the long-tailed distribution of available notes for training. Of the thirty DC3 unique cases, twenty-eight had at least one hundred document representatives across PubMed abstracts; we used these twenty-eight cases for our main experiments across models for consistency.

In our preliminary experiments, we hypothesized that the robustness of CliniqIR will likely increase as we consider diagnoses whose document frequency across our PubMed abstract collection falls within a certain threshold. Therefore, we tested this hypothesis within six PubMed document frequency thresholds across the MIMIC-III and DC3 datasets. (Figure S5) shows the class distribution frequency of MIMIC-III concept IDs (diagnoses) across the 33 million PubMed abstracts. (Figure S6) also shows the class distribution frequency of DC3 concept IDs (diagnoses) across the 33 million PubMed abstracts. The effect of PubMed coverage on CliniqIR models’ performance for the DC3 dataset is shown in (Figure S7) while (Figure S8) shows that the model’s performance on MIMIC-III increased with increasing PubMed coverage.


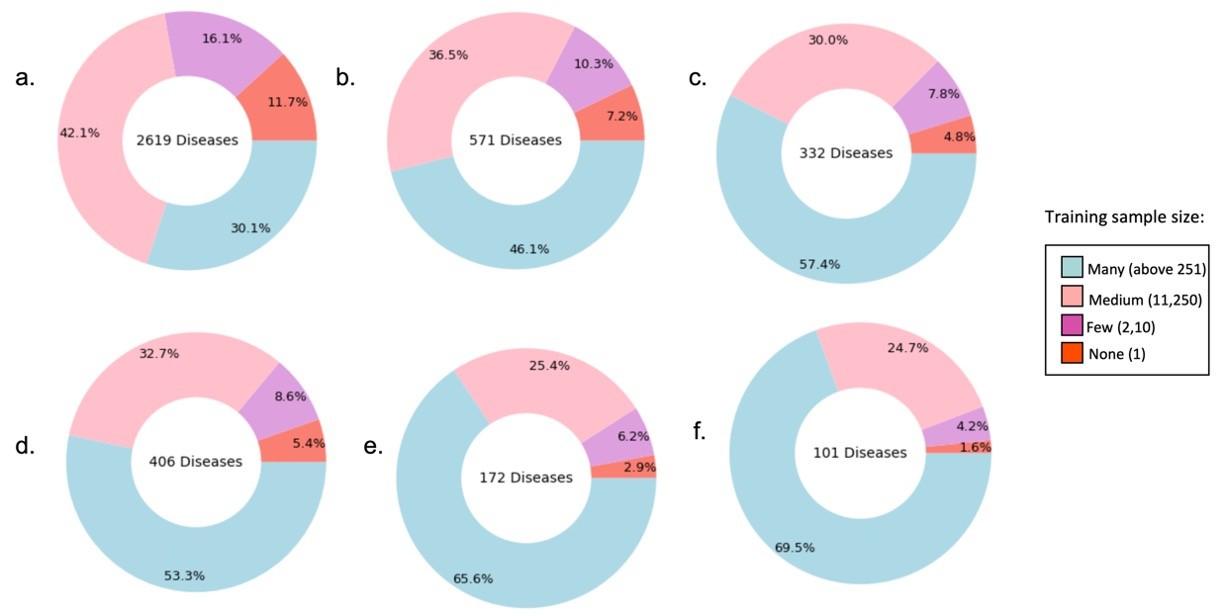
Figure S5: Class distribution of MIMIC-III diagnoses when their document frequency across the PubMed literature is (a) not considered, (b) above 99 (c) above 499, (d) above 999, (e) above 4,999 and (f) above 9,999. Legend indicates class sample size as well as training sample size. As we increase the document frequency threshold, the number of MIMIC-III diagnoses mentioned at least that many times in PubMed literature declines. For example, out of 2619 diagnoses, only 101 diagnoses had at least 10,000 document representations in the PubMed literature.


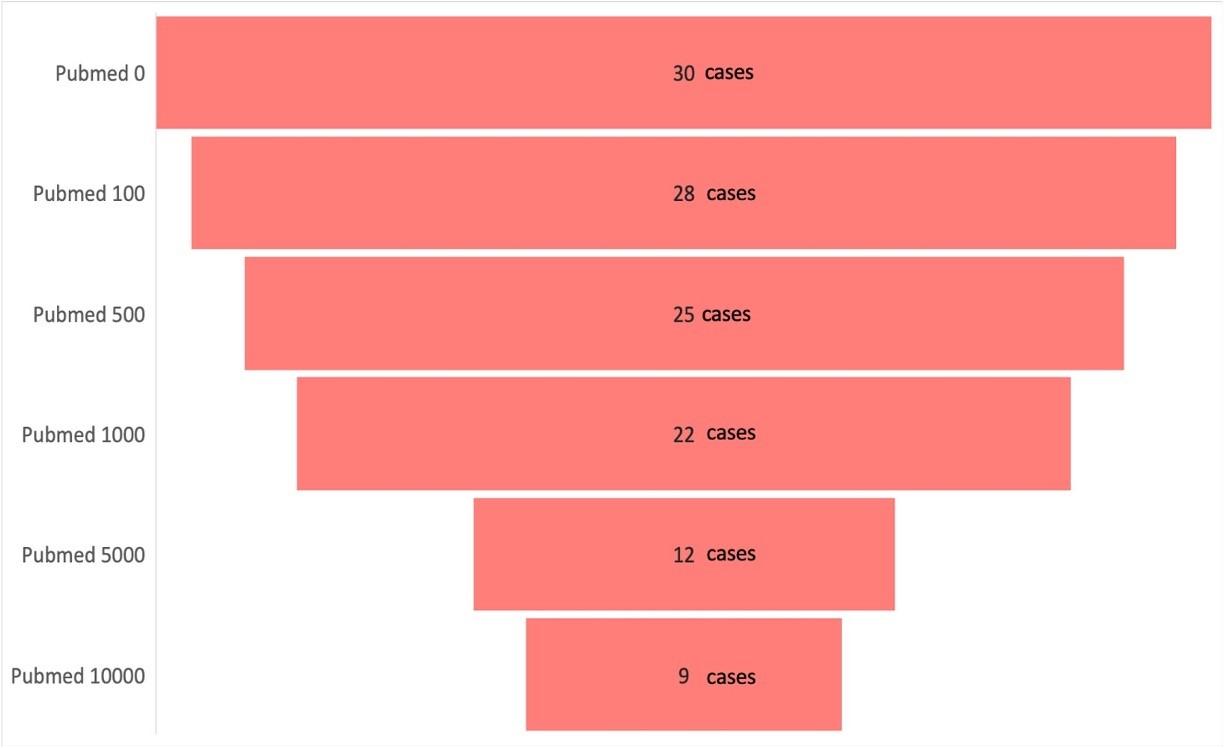


Figure S6: Number of unique cases across the DC3 datasets when their document frequency across the PubMed literature is not considered (PubMed 0), above 99 (PubMed 100), above 499 (PubMed 500), above 999 (PubMed 1000), above 4,999 (PubMed 5000) and above 9,999 (PubMed 10000). The cases across the DC3 datasets are unique; hence, the color of the bars indicates that the cases include no training sample. As we increase the document frequency threshold, the number of unique cases mentioned at least that many times in PubMed literature declines.


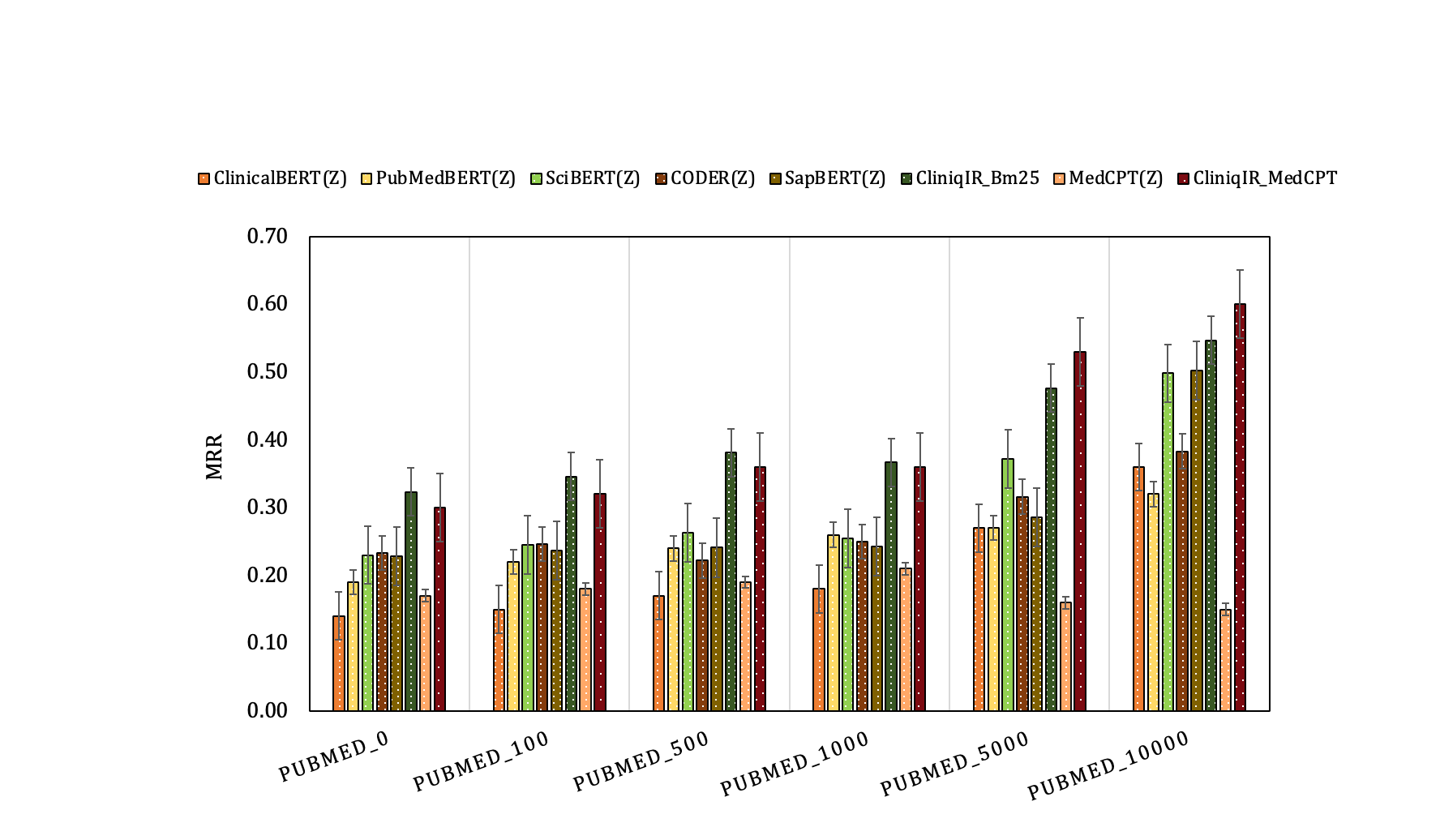


Figure S7: Performance evaluation of CliniqIR and the Zero-shot baselines across different dataset categories on the DC3 datasets. Each dataset category indicates the document frequency threshold of each diagnosis across PubMed. The performance of CliniqIR increases as the PubMed frequency threshold increases. CliniqIR outperforms all the zero-shot baselines across all dataset categories.

Figure S8: Performance evaluation of the models across different dataset categories on the MIMIC-III datasets. Each dataset category indicates the document frequency threshold of each diagnosis across PubMed. The performance of CliniqIR increases as the PubMed frequency threshold increases. In addition, the ensemble model gets better as the performance of CliniqIR increases.


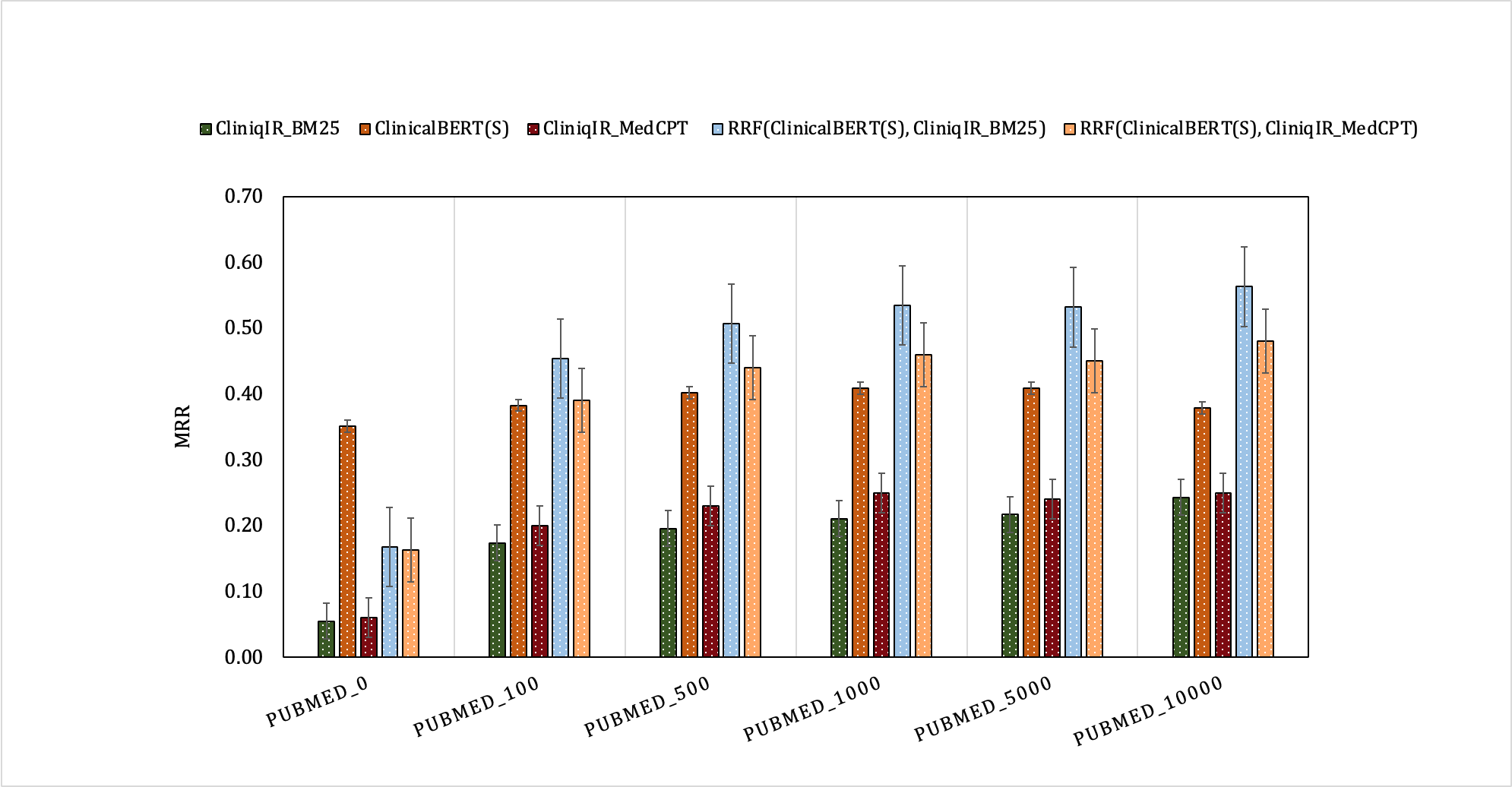


Figure S8: Performance evaluation of the models across different dataset categories on the MIMIC-III datasets. Each dataset category indicates the document frequency threshold of each diagnosis across PubMed. The performance of CliniqIR increases as the PubMed frequency threshold increases. In addition, the ensemble model gets better as the performance of CliniqIR increases.

# S6 Mean average precision on the DC3 and MIMIC-III datasets

Unlike the DC3 datasets, where the ground truth diagnosis could encompass multiple options, the MIMIC-III dataset contained only one definitive ground truth for each case. However, we still calculated the Mean Average Precision (MAP) metric for the retrieval models, CliniqIR_BM25 and CliniqIR_MedCPT, to better understand their retrieval capabilities. This is because MAP considers the entire ranking of retrieved documents, providing insights beyond just the top prediction. Conversely, calculating MAP for the supervised and zero-shot models wasn't necessary as these models already provided all possible ground truths before ranking the data. Figures S6 and S7 showcase their performance results using alternative metrics instead.


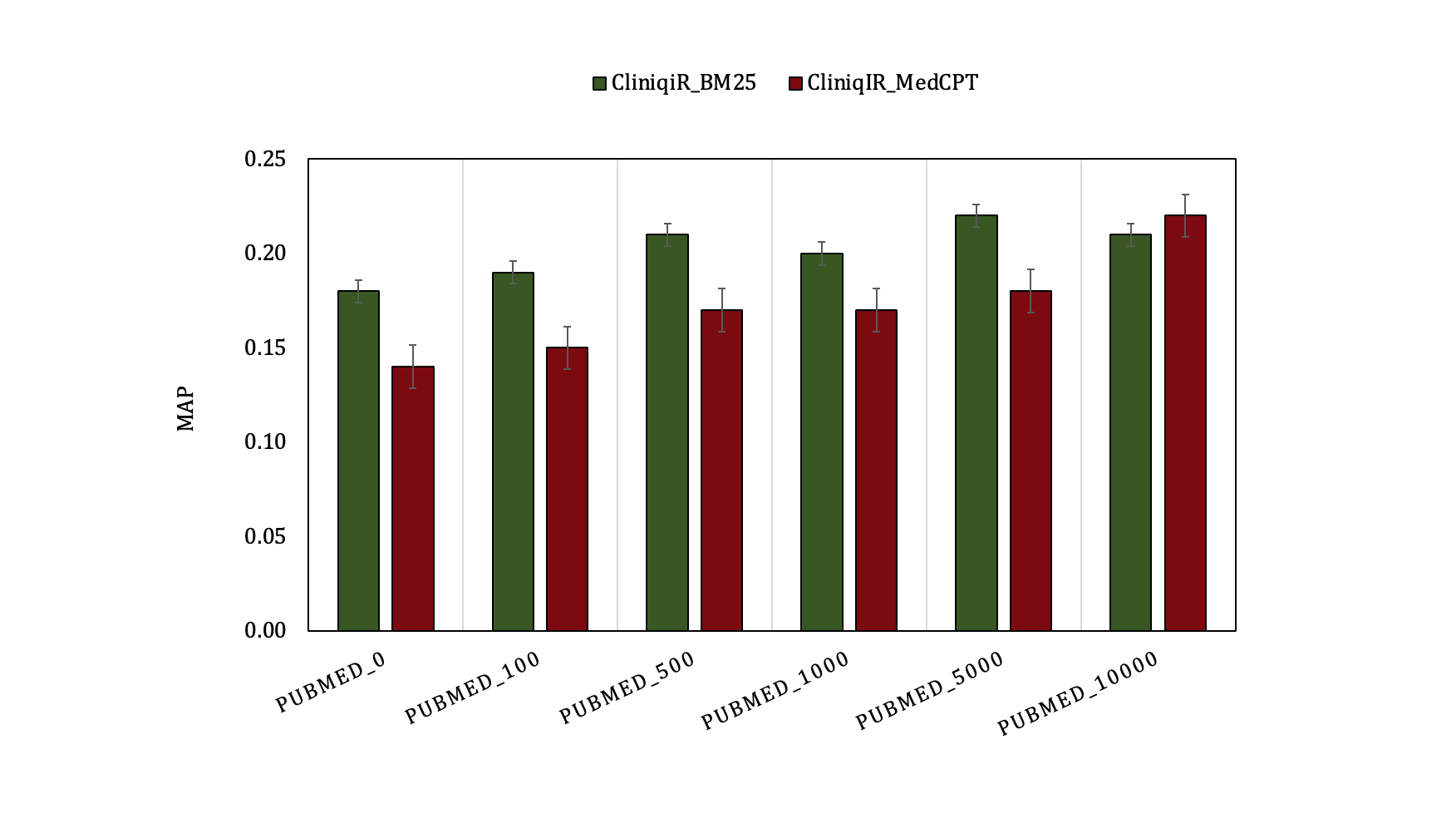


Figure S9: MAP performance evaluation of CliniqIR_BM25 and CliniqIR_MedCPT across different dataset categories on the DC3 datasets. Each dataset category indicates the document frequency threshold of each diagnosis across PubMed. The MAP performance of both models increases as the PubMed frequency threshold increases. CliniqIR_BM25 outperforms CliniqIR_MedCPT in most dataset categories.


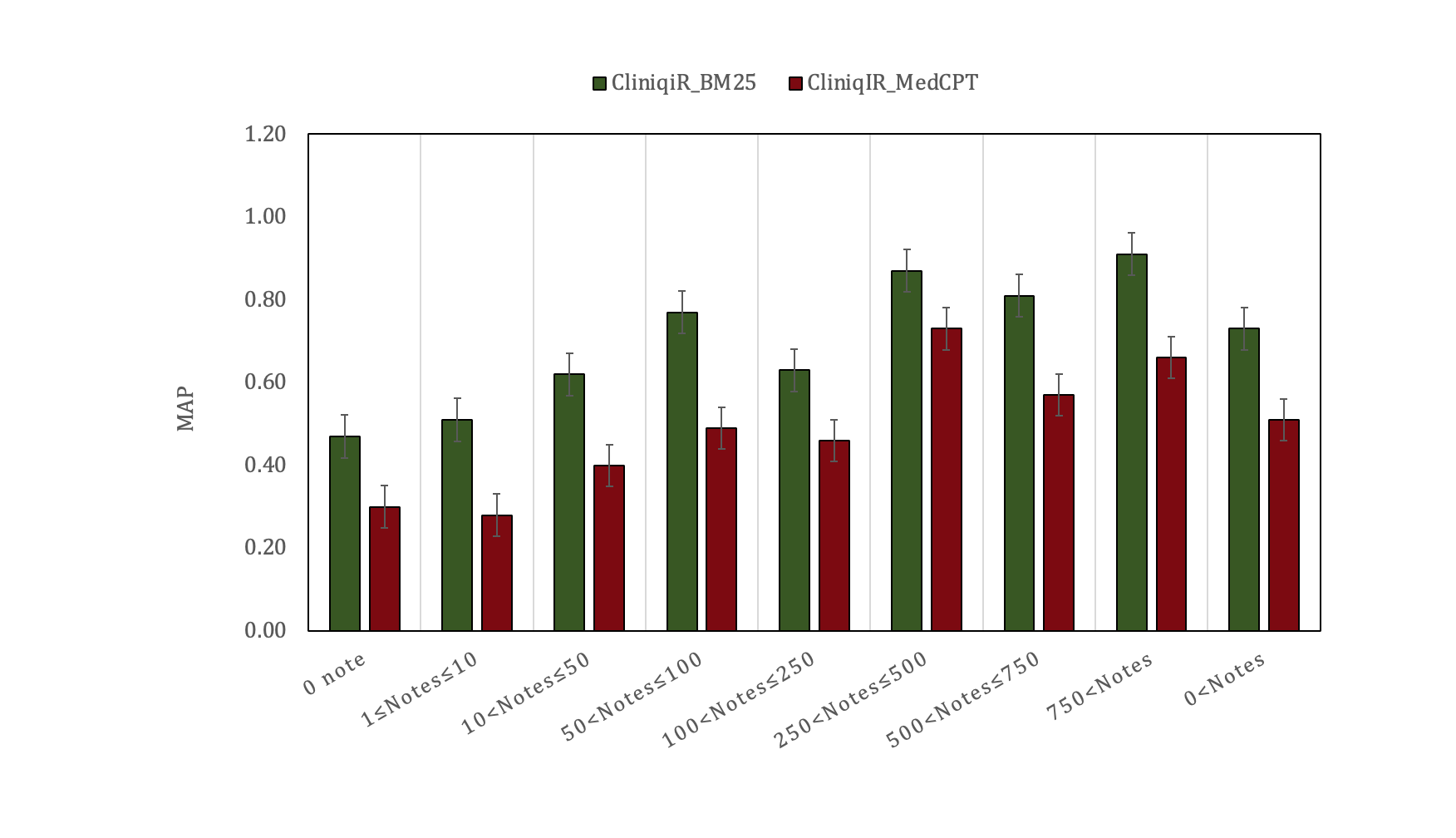


Figure S10: MAP performance evaluation of CliniqIR_BM25 and CliniqIR_MedCPT across different dataset categories on the MIMIC-III datasets. We categorized the results by the frequency of note representative per diagnosis. The MAP performance of both models increases as the PubMed frequency threshold increases. CliniqIR_BM25 outperforms CliniqIR_MedCPT in most dataset categories.


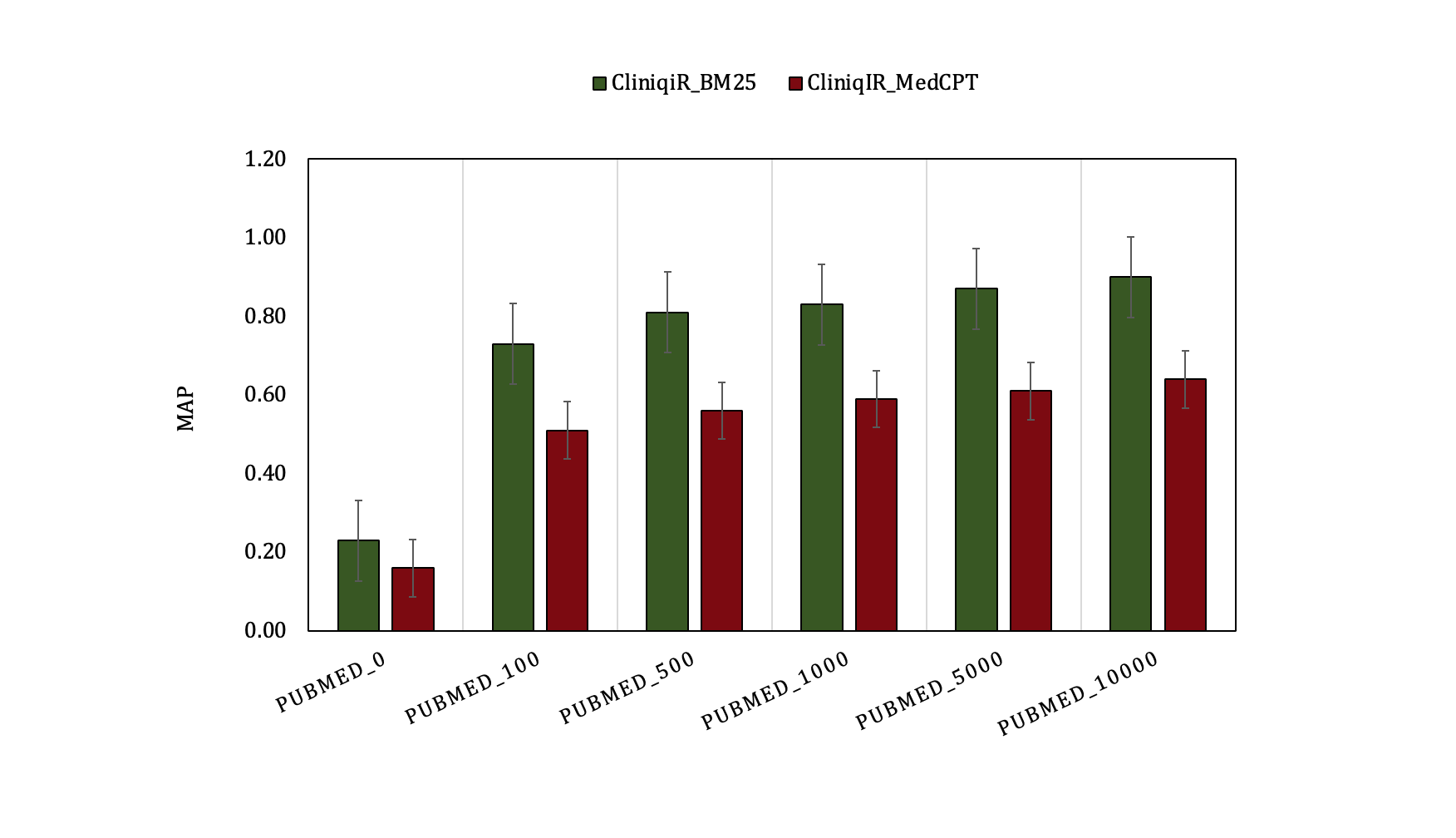
Figure S11: MAP performance evaluation of CliniqIR_BM25 and CliniqIR_MedCPT across different dataset categories on the MIMIC-III datasets. Each dataset category indicates the document frequency threshold of each diagnosis across PubMed. The MAP performance of both models increases as the PubMed frequency threshold increases. CliniqIR_BM25 outperforms CliniqIR_MedCPT in all dataset categories.
